# A Systematic Review and Meta-Analysis of Human Milk Feeding and Morbidity in Very Low Birth Weight Infants

**DOI:** 10.3390/nu10060707

**Published:** 2018-05-31

**Authors:** Jacqueline Miller, Emma Tonkin, Raechel A. Damarell, Andrew J. McPhee, Machiko Suganuma, Hiroki Suganuma, Philippa F. Middleton, Maria Makrides, Carmel T. Collins

**Affiliations:** 1 Healthy Mothers, Babies and Children, South Australian Health and Medical Research Institute, Adelaide, SA 5006, Australia; jacqueline.miller@sahmri.com (J.M.); andrew.mcphee@sa.gov.au (A.J.M.); machiko.suganuma@sahmri.com (M.S.); hiroki.suganuma@sahmri.com (H.S.); philippa.middleton@sahmri.com (P.F.M.); Maria.makrides@sahmri.com (M.M.); 2 Nutrition and Dietetics, Flinders University, Adelaide, SA 5001, Australia; emma.tonkin@flinders.edu.au (E.T.); raechel.damarell@flinders.edu.au (R.A.D.); 3 Neonatal Medicine, Women’s and Children’s Hospital, Adelaide, SA 5006, Australia; 4 Adelaide Medical School, Discipline of Paediatrics, The University of Adelaide, Adelaide, SA 5006, Australia

**Keywords:** preterm infant, human milk, necrotising enterocolitis, sepsis, bronchopulmonary dysplasia, retinopathy of prematurity, neurodevelopment, donor human milk, formula feeding

## Abstract

This systematic review and meta-analysis synthesised the post-1990 literature examining the effect of human milk on morbidity, specifically necrotising enterocolitis (NEC), late onset sepsis (LOS), retinopathy of prematurity (ROP), bronchopulmonary dysplasia (BPD) and neurodevelopment in infants born ≤28 weeks’ gestation and/or publications with reported infant mean birth weight of ≤1500 g. Online databases including Medline, PubMed, CINAHL, Scopus, and the Cochrane Central Register of Controlled Trials were searched, and comparisons were grouped as follows: exclusive human milk (EHM) versus exclusive preterm formula (EPTF), any human milk (HM) versus EPTF, higher versus lower dose HM, and unpasteurised versus pasteurised HM. Experimental and observational studies were pooled separately in meta-analyses. Risk of bias was assessed for each individual study and the GRADE system used to judge the certainty of the findings. Forty-nine studies (with 56 reports) were included, of which 44 could be included in meta-analyses. HM provided a clear protective effect against NEC, with an approximate 4% reduction in incidence. HM also provided a possible reduction in LOS, severe ROP and severe NEC. Particularly for NEC, any volume of HM is better than EPTF, and the higher the dose the greater the protection. Evidence regarding pasteurisation is inconclusive, but it appears to have no effect on some outcomes. Improving the intake of mother’s own milk (MOM) and/or donor HM results in small improvements in morbidity in this population.

## 1. Introduction

Human milk (HM) is the feed of choice for preterm infants [1]. However, not all mothers can provide sufficient milk to meet requirements, and supplementation with either preterm formula (PTF) or donor human milk (DHM) is common practice. Current recommendations are for the use of mother’s own milk (MOM), when available, with appropriately screened and pasteurised DHM the next best choice if there is insufficient MOM [2,3]. Some countries, such as Norway, have largely used unpasteurised DHM, which may contribute to the protection DHM provides [4]. Previous randomised trials (RT) [5,6,7,8,9,10,11,12,13,14] and meta-analyses [15,16], provide evidence of the relative advantages of HM feeding compared with formula feeding. Advantages of HM feeding, with either pasteurised DHM or MOM, include protection from necrotising enterocolitis (NEC) [16,17] and infection [11,14] and improved cognitive outcomes, with very low birth weight (VLBW) infants having the greatest advantage [15]. However, much of this early research reflects feed compositions, clinical management and technology available in the 1980s and is now outdated. Advances in many non-nutrition related aspects of care, particularly the introduction of surfactant in the early 1990s represented a significant advance in the care of preterm infants with dramatic reduction in mortality and morbidity [18]. In addition, human milk banks, while common in some countries, are beginning to re-emerge in other countries, such as Australia, where access to DHM is limited and prioritised for the most vulnerable infants who stand to gain the most benefit. Currently in Australia there are now five milk banks in operation [19]. Therefore, an estimated 75% of Australian high risk preterm infants do not have access to pasteurised DHM. This systematic review summarises evidence on associations between different modes of feeding and morbidity. It aims to provide a direct comparison between exclusive human milk (EHM) and exclusive preterm formula (EPTF), to examine whether any HM was protective when infants also receive preterm formula and to explore the dose related effect of HM. As pasteurisation is known to destroy some of the immune-protective properties of HM [20,21,22] and unpasteurised MOM to provide antibodies specific to the mother–infant dyad [23], we also aimed to determine the effects of pasteurization of HM on infant morbidity.

Is DHM important for the preterm infant whose mother provides enough breast milk to meet nearly all their requirements? Should it be limited to those who are unable to provide any HM? Is there a dose that achieves greatest clinical benefit? We therefore reviewed RTs and observational studies that examined the effect of HM on preterm infant morbidity. 

## 2. Methods

### 2.1. Registration

The review is registered with PROSPERO International prospective register of systematic reviews and the protocol is available from [24]. 

### 2.2. Eligibility Criteria

#### 2.2.1. Types of Studies

Experimental and observational studies, published from 1990 onwards, were considered for inclusion in this review. All component studies of relevant systematic reviews were also considered.

#### 2.2.2. Types of Participants

Infants born ≤28 weeks’ gestation (from 1990 onwards) and/or publications with reported study population mean birth weight of ≤1500 g were considered for inclusion. Post-discharge feeding studies were excluded. As quantifying the exposure (HM) was necessary to group studies, we excluded any studies where this could not be done reliably, e.g. where feeding intake data were reported retrospectively or measured at only one time point and extrapolated for the neonatal admission.

#### 2.2.3. Types of Intervention

Studies comparing the effects of HM were grouped according to the following exposure categories:

1. EHM compared with EPTF—to provide a direct evaluation of the benefits of an exclusive HM diet.
2. Any HM (includes EHM or HM plus preterm formula) compared with EPTF—to examine whether any HM was protective when infants also receive preterm formula.
3. Dose related—exclusive or higher HM (higher dose HM plus preterm formula) intake compared with lower HM (lower dose HM plus preterm formula) intake. This comparison was included to explore the dose related effect of HM, i.e., is more HM beneficial when infants have at least some HM? No a priori categories were defined for “higher” or “lower” dose of HM. Therefore, studies included here may have compared EHM with mixed feeding groups, or alternatively all infant groups may have been mixed fed, with those having a higher proportion of enteral intake as HM compared with those having a lower proportion of enteral intake as HM.
4. Unpasteurised HM compared with pasteurised HM (DHM and/or MOM). Pasteurisation is known to destroy some of the immune-protective properties of HM [20,21,22] and unpasteurised MOM provides antibodies specific to the mother–infant dyad [23]. Thus, this comparison was included to assess the effect of pasteurisation on the potential benefits of HM, and to attempt to differentiate the benefits associated with any HM, and those only associated with unpasteurised MOM. Studies reporting mixed feeding (HM plus PTF) were included if there was also a comparison between pasteurised and unpasteurised HM.

#### 2.2.4. Type of Outcome Measures

Outcomes included NEC (any and severe), late onset sepsis (LOS), bronchopulmonary dysplasia (BPD), retinopathy of prematurity (ROP) (any and severe), and neurodevelopment, defined as cognitive or motor development. A holistic approach to inclusion was taken, therefore no single definition for each outcome was predetermined. Studies reporting composite outcomes (e.g., incidence of NEC and death) were excluded unless data were also provided for each outcome separately.

### 2.3. Information Sources and Study Selection

Primary papers and systematic reviews were identified by searching databases including: Medline (Ovid), PubMed, CINAHL (EBSCOhost), Scopus, and the Cochrane Central Register of Controlled Trials (Wiley). All searches were conducted on 13 June 2017. Results were restricted to English language studies published from 1990.

The search strategy incorporated and combined three key concepts: preterm infant, human milk feeding, and the specific morbidity outcomes of interest to the review. Each concept search employed a wide range of synonyms to ensure maximum retrieval of relevant literature. The searches also used database-specific subject headings (e.g., MeSH terms in Medline) where available. The search strategies are provided as Supplementary Materials (Tables S1 and S2). The review authors checked the reference lists of relevant articles to ensure literature saturation. Citations were exported to Covidence [25] for organisation and screening. Two authors independently screened articles against the eligibility criteria by title and abstract. Full texts of articles were then retrieved, and two authors independently screened for inclusion. Disagreements were resolved by discussion between two authors; if no agreement could be reached, a third author would decide. The PRISMA diagram showing selection of studies is provided as Supplementary Materials (Figure S1).

### 2.4. Data Extraction, Risk of Bias in Individual Studies and Data Synthesis

Two authors extracted the data into tables. Study designs were classified according to the Cochrane Effective Practice and Organisation of Care (EPOC) [26]. Risk of bias for RTs was assessed using the Cochrane Risk of Bias tool [27] and Critical Appraisal Skills Programme checklists [28] for other study designs. Each study was assessed for selection, attrition and reporting bias and assessed as low, medium or high risk of bias.

Where possible, results of RTs and observational studies were separately included in a meta-analysis, using Review Manager (RevMan), Version 5.3, 2014 [29]. For dichotomous outcomes, results were expressed as risk ratios (RR) with 95% confidence intervals (CI). Neurodevelopmental outcomes were pooled, provided the test scale was standardised, and expressed as mean difference (MD) with 95% CI. A random-effects model was used (and is the model applied in all forest plots). Where statistical heterogeneity was low to moderate (*I*^2^ ≤ 50%), a fixed effects model was used and where this changed statistical significance this has been noted in text. Where possible we have explained statistical heterogeneity above 50%.

A “Summary of findings” table was prepared for each comparison using the GRADE system (GRADEpro GDT, 2015) [30]. GRADE is designed to evaluate the quality of evidence and strength of recommendations. RTs with no limitations are considered high quality evidence and observational studies as providing low quality evidence. Studies can then be downgraded by one (for serious concern) or two (for very serious concerns) based on risk of bias, inconsistency, indirectness, imprecision and publication bias. Observational studies with a large effect size have been upgraded by one for a strong association, defined as a RR of ≤0.5 [31]. For each outcome, we report our certainty in the findings as very low, low, moderate or high separately according to study design (RTs, observational).

To interpret the overall evidence for each outcome and comparison, we used the following terminology:

1. Clear effect/clear evidence of no effect: The certainty of evidence is moderate or above with a clinically important result from RTs, ideally aligning with results from observational studies or moderate certainty evidence from observational studies; and with reasonable numbers of events and/or participants.
2. Probably an effect/probably no effect: There is moderate certainty from either RTs or observational studies and point estimates may be different between the 2 study types with overlapping CIs but can be explained (e.g., through heterogeneity). There are large numbers of participants and studies.
3. Possible effect/possibly no effect: There is low/ moderate certainty with CIs which may suggest a difference although not reaching conventional statistical significance; or with a confidence interval which indicates a trivial difference only.
4. Inconclusive: The certainty of evidence is very low to low, CIs are wide, and number of participants and studies is low.

Where possible the overall effect (absolute risk reduction (ARR), or mean difference (MD), with 95% CI) have been reported.

Table 1 provides details of the included studies. Figures showing forest plots for all outcomes are presented in the manuscript, except for severe NEC and severe ROP which are presented in the Supplementary Materials (Figures S2 and S3). Individual summary of findings tables for each comparison and outcome are presented in the Supplementary Materials (Tables S3–S9) with a collated summary of findings table presented in the manuscript (Table 2).

## 3. Results

The search and selection processes are described in Figure S1. Forty-nine unique studies (with 56 reports) were identified for this review and included 6 RTs [32,33,34,35,36,37], 1 non-randomised intervention trial [38], 27 cohort (with 32 reports) [22,23,39,40,41,42,43,44,45,46,47,48,49,50,51,52,53,54,55,56,57,58,59,60,61,62,63,64,65,66,67,68], 7 interrupted time series [20,21,69,70,71,72,73], and 8 case-control studies (with 10 reports) [74,75,76,77,78,79,80,81,82,83]. Forty unique studies (with 44 reports) could be included in meta-analyses.

### 3.1. Risk of Bias

The six RTs [32,33,34,35,36,37] all had low risk of bias (Table 1). Sequence generation was not reported in three [34,36,37], blinding was not possible in one [37] and blinding of physicians but not nursing staff occurred in another [33]. However, as the outcomes of interest are objective, we thought these unlikely to introduce bias. Of the observational studies, 26 were assessed as low risk of bias, 14 as moderate and 3 as high (Table 1).

### 3.2. Necrotising Enterocolitis (NEC)

NEC was reported in 42 studies and severe NEC, defined as NEC requiring surgery, in 12 [23,32,33,34,37,41,49,63,64,66,70,76]. NEC was defined by most as Bell’s stage 2 or higher, with 6 defining by clinical signs and or radiological evidence [37,38,55,61,63,65] and 8 studies [22,23,40,49,50,54,60,71] providing no definition (Table 1).

#### 3.2.1. Comparison 1: Exclusive Human Milk vs. Exclusive Preterm Formula

**Figure 1:**
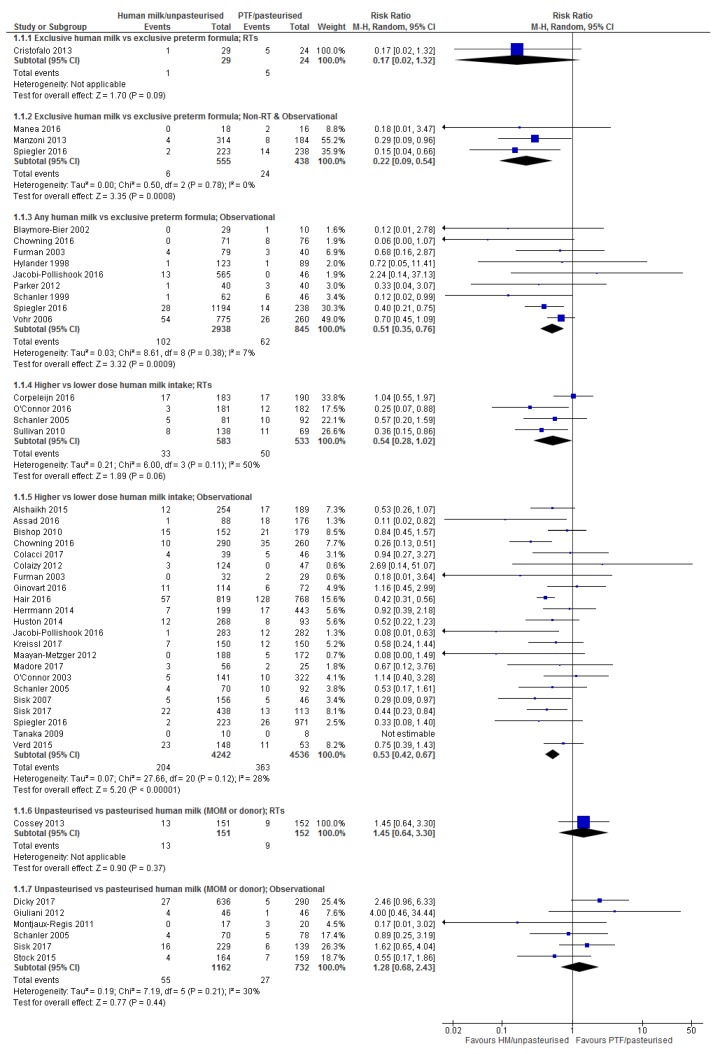
Forest plot of relative risk for the association between human milk and necrotising enterocolitis.

Randomised Trials: One small RT [34] (Table 1) reported the effect of EHM, including a human milk derived fortifier, versus EPTF, on the risk of NEC (any and severe) and did not detect a difference (any NEC RR 0.17, 95% CI 0.02, 1.32; *n* = 53, Figure 1, low certainty, Table S3; severe NEC RR 0.09, 95% CI 0.01, 1.64; *n* = 53, Figure S2, low certainty, Table S4).

Observational studies: Two cohort [58,66] and one non-randomised studies [38] reported this comparison for any NEC (Table 1) and on meta-analysis showed a significant reduction in any NEC (RR 0.22, 95% CI 0.09, 0.54, *n* = 933; *I*^2^ 0%; Figure 1; moderate certainty, Table S3).

Severe NEC was reported in one cohort study [66] that did not detect a difference (RR 0.22, 95% CI 0.03, 1.86, *n* = 444; Figure S2; low certainty, Table S4).

Overall: The observational studies show there is a possible reduction in any NEC with EHM compared with EPTF (ARR, 4.3%, from 2.5 to 5 fewer cases/100) (Table 2). There is inconclusive evidence relating to severe NEC (Table S4).

#### 3.2.2. Comparison 2: Any Human Milk vs. Exclusive Preterm Formula

Randomised trials: There were no RTs identified reporting NEC for this comparison.

Observational studies: Nine cohort studies [40,41,47,52,54,61,63,66,68], comparing infants fed any HM with infants fed EPTF (Table 1), were included in the meta-analysis for this comparison (Figure 1) There was a clear effect of any HM in reducing NEC (RR 0.51, 95% CI 0.35, 0.76, *n* = 3783, *I*^2^ 7%; Figure 1; moderate certainty, Table S3). Henderson et al. [77] reported a case controlled study (53 NEC cases, 53 controls) from 10 NICUs in the UK which could not be included in the meta-analysis. Seventy-five percent of NEC cases received any HM compared with 91% of controls (OR 0.32, 95% CI 0.11, 0.98). This finding is consistent with the meta-analysis, however all stages of NEC were included (whereas most other studies defined NEC as Bell’s stage 2 or above), and matched controls on GA only, which may not have accounted for other potential confounders [77].

Severe NEC was reported in three cohort studies [41,63,66] with no difference detected (RR 0.30, 95% CI 0.05, 1.76, *n* = 1420, *I*^2^ 50%; Figure S2; low certainty, Table S4).

Overall: There is a clear effect of any HM in reducing NEC (any) with an ARR of 3.6% (from 1.8 to 4.8 fewer cases/100); the evidence is inconclusive for severe NEC (Table 2).

#### 3.2.3. Comparison 3: Higher vs. Lower Dose Human Milk Intake

Randomised trials: Four RTs [32,35,36,37] (Table 1) were included in the meta-analysis for this comparison (Figure 1) and showed a reduction in any NEC (RR 0.59, 95% CI 0.39, 0.89, *n* = 1116; fixed effects; Figure 1; moderate certainty, Table S3). In all four trials the higher dose of HM was a combination of MOM and DHM, therefore making this an EHM group while HM intake in the low dose group was either not reported [36] or varied between a median proportion of enteral intake of 63% [35] to 85% [32].

Two RTs [32,37] reported severe NEC (Table 1) and showed no difference between higher vs. lower dose of HM (RR 0.36, 95% CI 0.06, 2.04, *n* = 580, *I*^2^ 66%; Figure S2, low certainty, Table S4). A possible explanation for the heterogeneity is the use of a human milk derived fortifier (HMDF) by Sullivan et al. [37] but not by Corpeleijn et al. [32].

Observational studies: Twenty-two observational studies (16 cohort studies [22,23,41,42,43,47,49,51,54,55,56,60,64,65,66,73], 5 interrupted time series [21,69,70,71,72], and 1 non-randomised arm of an intervention trial [36]) were included in the meta-analysis for this comparison (Table 1) which showed a significant reduction in any NEC (RR 0.53, 95% CI 0.42, 0.67, *n* = 8778; *I*^2^ 28%; Figure 1; moderate certainty, Table S3).

Meta-analysis (Figure S1) of the six studies that reported severe NEC [23,41,49,64,66,70] (Table 1) showed a reduction with a higher dose of HM (RR 0.51, 95% CI 0.33, 0.79, *n* = 2964, *I*^2^ 0%; Figure S2; moderate certainty, Table S4).

An additional three case-control studies [74,78,79] addressed this comparison but could not be included in the meta-analysis (Table 1). Kimak et al. [79] included 55 NEC cases and 973 controls matched by birth weight category. The odds of developing NEC were four times higher if the duration of exclusive HM feedings was <7 days, compared with ≥7 days (OR 4.02, 95% CI 1.23, 13.11). Johnson et al. [78] (29 NEC cases, 262 controls) showed no clear effect of HM dose on NEC development (mean, SD, intake of HM day 1–14 NEC cases 26 ± 18 vs. controls 30 ± 28 mL/kg/day, *p* = 0.25). Bensouda et al. [74] (38 NEC cases, 76 controls) showed that fewer NEC cases received breastmilk (63% vs. 87%, *p* = 0.02). These studies align with our meta-analysis results.

Overall: There is a clear reduction in the incidence of any NEC with higher dose HM (ARR ranging from 4.3% (0.2 more to 6.8 fewer cases/100 for RTs to 3.8% (2.6 to 4.6 fewer cases/100) for observational studies) (Table 2). There is a possible reduction in the incidence of severe NEC (ARR from the observational studies 1.8%, from 0.8 to 2.4 fewer cases/100) (Table 2).

#### 3.2.4. Comparison 4: Unpasteurised vs. Pasteurised Human Milk

Randomised trials: One RT [33] assessed the effect of unpasteurised compared with pasteurised HM on any and severe NEC (Table 1) and did not detect a difference in either (any NEC RR 1.45, 95% CI 0.64, 3.3, *n* = 303; Figure 1, low certainty, Table S3; or severe NEC RR 0.11, 95% CI 0.01, 2.06, *n* = 303; Figure S2, low certainty, Table S4).

Observational studies: Six observational studies (three cohort [44,59,64], one interrupted time series [20] one case-control [76] and one non-randomised arm of an intervention trial [36]) (Table 1) were included in the meta-analysis and did not detect a difference in any NEC between unpasteurised and pasteurised HM (RR 1.28, 95% CI 0.68, 2.43, *n* = 1894, *I*^2^ 30%; Figure 1; low certainty, Table S3). Two of these studies reported severe NEC and did not detect a difference (RR 1.59, 95% CI 0.14, 17.85, *n* = 530, *I*^2^ 42%; Figure S2; low certainty, Table S4).

Overall: The evidence for an effect of pasteurised vs. unpasteurised HM on the incidence of any or severe NEC is inconclusive (Table 2).

### 3.3. Late Onset Sepsis (LOS)

LOS was reported in 35 studies with the majority (*n* = 23) defining sepsis by the presence of a positive blood culture at >48 h to >5 days, with the need for supportive laboratory markers, treatment with antibiotics and for multiple positive cultures in the case of coagulase negative *Staphylococcus*. In the remaining 12 studies, the diagnosis was either based on clinical markers [20,36,37,58] or not defined [21,40,49,51,54,60,65,73] (Table 1).

#### 3.3.1. Comparison 1: Exclusive Human Milk vs. Exclusive Preterm Formula

**Figure 2:**
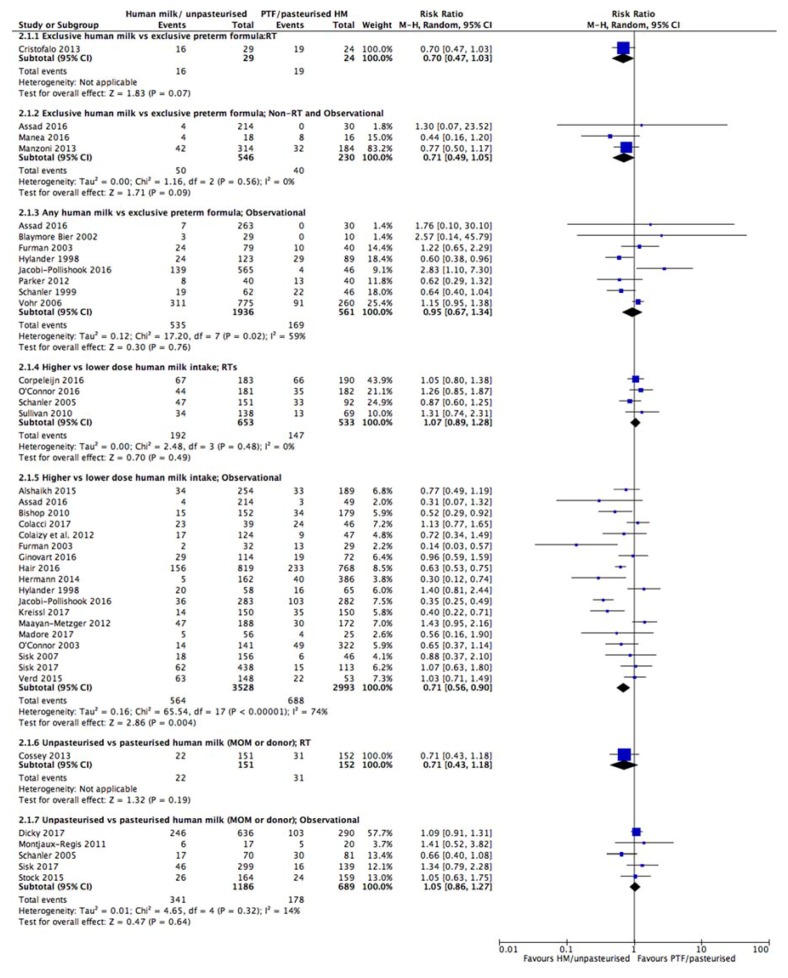
Forest plot of relative risk for the association between human milk and late onset sepsis.

Randomised trials: One RT [34] reported the effect of EHM feeding (including a human milk derived fortifier) compared with EPTF feeding on the incidence of LOS (Table 1) and indicating a possible reduction in LOS (RR 0.70, 95% CI 0.47, 1.03; *n* = 53, Figure 2; low certainty, Table S5).

Observational studies: Three observational studies (one non-randomised trial [38], one interrupted time series [21] and one cohort study [58]) (Table 1) were included in the meta-analysis for this outcome. There was a possible reduction in LOS with EHM feeding (RR 0.71, 95% CI 0.49, 1.05; *n* = 776, *I*^2^ 0%, Figure 2; low certainty, Table S5).

Overall: Although the RT and meta-analysis of observational studies did not reach significance, the CIs neared 1 and as such, thus we conclude there is a possible reduction in the incidence of LOS with an EHM diet (ARR from RT of 23.8% (from 42 fewer to 2.4 more cases/100) and from observational studies 5% (from 0.9 more to 8.9 fewer cases/100; Table 2).

#### 3.3.2. Comparison 2: Any Human Milk vs. Exclusive Preterm Formula

Randomised trials: There were no RTs identified reporting LOS for this comparison

Observational studies: Eight observational studies including seven cohort [40,47,52,54,61,63,68], and one interrupted time series study [21] (Table 1) compared the incidence of LOS in infants fed with any HM compared with those fed exclusively with PTF. On meta-analysis, no difference was detected on LOS (RR 0.95, 95% CI 0.67, 1.34; *n* = 2497, *I*^2^ 59%, Figure 2, very low certainty, Table S5). The source of the heterogeneity is not readily apparent but baseline differences in the population and varying dosage of HM may contribute. 

Overall: The evidence to determine if the receipt of any HM compared with EPTF reduces LOS is inconclusive (Table 2).

#### 3.3.3. Comparison 3: Higher vs. Lower Dose Human Milk Intake

Randomised trials: The impact of high dose vs. low dose HM on the incidence of LOS was addressed in five RTs, four of which could be combined in a meta-analysis [32,35,36,37] (Table 1, Figure 2). No difference in LOS was detected with higher vs. lower dose HM (RR 1.07, 95% CI 0.89, 1.28, *n* = 1186, *I*^2^ 0%, Figure 2; moderate certainty, Table S5). In contrast, Cossey et al. [33] reported the risk of LOS according to quantity of human milk, in increments of 10 mL/kg/day, and showed that the risk of LOS was lower as both the quantity (hazard ratio (HR) 0.89, 95% CI 0.83, 0.95, *p* = 0.0008) and cumulative quantity of MOM increased over time (HR 0.99, 95% CI 0.98, 0.99, *p* = 0.0001).

Observational studies: Nineteen observational studies, 18 of which could be combined in a meta-analysis, reported this comparison (Table 1). They comprised six interrupted time series [21,69,70,71,72,73] and 12 cohort studies [23,42,43,47,49,52,54,55,56,60,64,65] and on meta-analysis showed a significantly lower incidence of infection in infants exposed to higher vs. lower human milk intakes (RR 0.71, 95% CI 0.56, 0.90, *n* = 6521 infants; *I*^2^ = 74%; Figure 2; very low certainty, Table S5). Heterogeneity is likely due to differences in study design and variation in the dose of HM in both the high and low groups.

A further prospective case-control study [82] conducted logistic regression and found an independent protective effect of the average daily dose of HM for every 10 mL/kg/day increase from day of life 1–28 (OR 0.98, 95% CI 0.97, 0.99, *p* = 0.008).

Overall: The evidence for high vs. low dose HM on reducing LOS from RTs and observational studies differs and is inconclusive (Table 2).

#### 3.3.4. Comparison 4: Unpasteurised vs. Pasteurised Human Milk

Randomised trials: One RT assessed the impact of unpasteurised HM vs. pasteurised HM on the risk of neonatal LOS [33] (Table 1) and showed no difference in the effect of pasteurisation on LOS (RR 0.71, 95% CI 0.43, 1.18, *n* = 303, Figure 2; moderate certainty, Table S5).

Observational studies: A meta-analysis of five studies, including three cohort studies [44,59,64], one interrupted time series [20] and one non-randomised arm of an RT [36] (Table 1) which compared the effect of unpasteurised vs. pasteurised milk on LOS showed no difference (RR 1.05, 95% CI 0.86,1.27, 1875 infants, *I*^2^ = 14%, Figure 2, low certainty, Table S5).

Overall: The use of unpasteurised compared with pasteurised human milk is not likely to have an effect on the incidence of LOS (Table 2).

### 3.4. Bronchopulmonary Dysplasia (BPD)

BPD was reported in 29 studies; the majority (*n* = 20) defined BPD as supplemental oxygen requirement and/or respiratory support at 36 weeks’ post menstrual age [21,33,35,36,37,42,44,47,54,56,59,60,64,66,69,70,71,76,78], four studies as supplemental oxygen requirement for at least 28 days [32,49,55,75], one as oxygen requirement at discharge [43] and a further four studies did not provide a definition [22,52,65,73] (Table 1). 

#### 3.4.1. Comparison 1: Exclusive Human Milk Compared with Exclusive Preterm Formula

Randomised trials: There were no RTs reporting BPD for this comparison.

**Figure 3:**
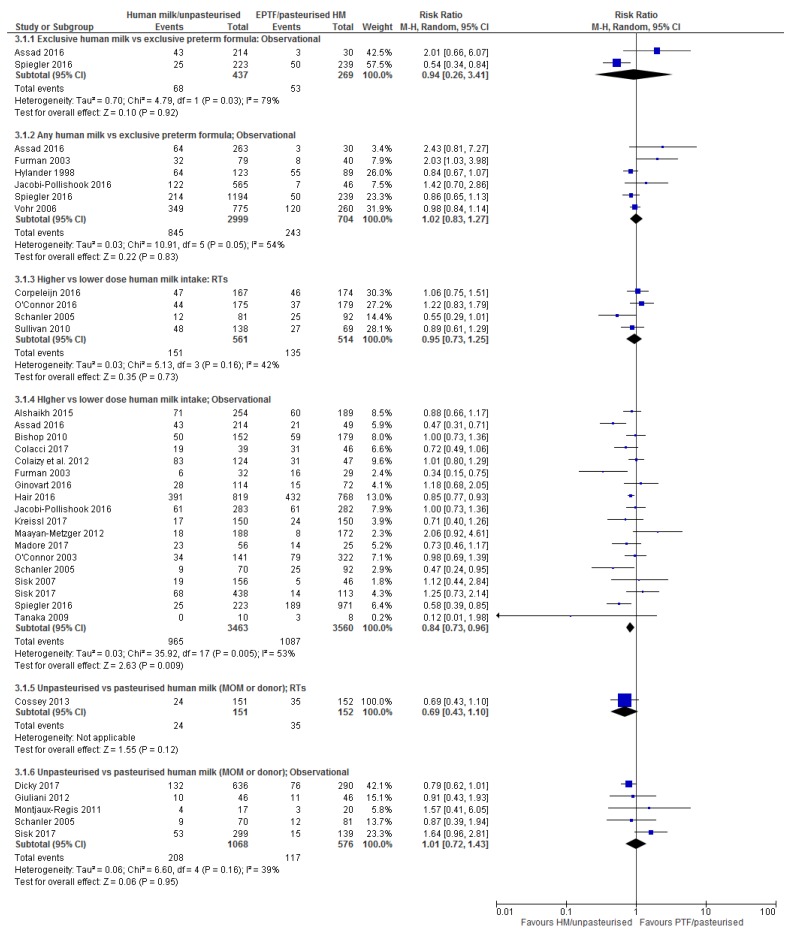
Forest plot of relative risk for the association between human milk and bronchopulmonary dysplasia.

Observational studies: The relationship between an EHM diet and EPTF diet on BPD was reported in two observational studies, an interrupted time series [21] and a cohort study [66] (Table 1). There was no effect of an EHM diet on BPD (RR 0.94, 95% CI 0.26, 3.41; *n* = 706; *I*^2^ = 79%, Figure 3; very low certainty, Table S6). Heterogeneity is possibly due to differences in study design. 

Overall: The evidence for an effect of EHM vs. EPTF on BPD is inconclusive (Table 2).

#### 3.4.2. Comparison 2: Any Human Milk Compared with Exclusive Preterm Formula

Randomised Trials: There were no RTs reporting BPD for this comparison.

Observational studies: Six studies (one interrupted time series [21] and five cohort [47,52,54,66,68]) reported BPD for this comparison (Table 1). On meta-analysis, no difference in BPD was detected (RR 1.02, 95% CI 0.83, 1.27; *n* = 3703; *I*^2^ = 54%, Figure 3; very low certainty, Table S6). Heterogeneity may be explained by baseline differences in GA and BW which may favour EPTF.

Overall: The evidence for an effect of any HM compared to EPTF on the incidence of BPD is inconclusive (Table 2).

#### 3.4.3. Comparison 3: Higher vs. Lower Dose Human Milk Intake

Randomised Trials: Four RTs reported the effect of this comparison on BPD [32,35,36,37] (Table 1). No difference on BPD was detected on meta-analysis (RR 0.95, 95% CI 0.73, 1.25; I^2^ = 42%, *n* = 1075, Figure 3; low certainty, Table S6).

Observational studies: Twenty studies (five interrupted time series [21,69,70,71,73], two case-control [75,83], twelve cohort [22,42,43,47,49,54,55,56,60,64,65,66] and one non-randomised arm of an RT [36]) (Table 1) reported BPD for this comparison. On meta-analysis of the 18 studies that could be included, there was a reduction in BPD associated with a higher dose of human milk (RR 0.84, 95% CI 0.73, 0.96, *n* = 7023; *I*^2^ = 53%, Figure 3; very low certainty, Table S6). 

Data from two case control studies [75,83] were unable to be included in the meta-analysis. Both studies showed a reduction in BPD associated with increasing amounts of human milk. Fonseca et al. [75] reported that a minimum amount of human milk (≥7 mL/kg/day) in the first 42 days was associated with a reduced incidence of BPD and Patel et al. [83] reported that, for every 10% increase in HM intake, the risk of BPD was reduced (RR 9.5%, 95% CI 0.824, 0.995).

Overall: The evidence for an effect of high vs. low dose HM on BPD is inconclusive (Table 2).

#### 3.4.4. Comparison 4: Unpasteurised vs. Pasteurised Human Milk

Randomised Trials: One RT reported the effect of unpasteurised vs. pasteurised MOM on BPD [33] (Table 1) with no effect demonstrated (RR 0.69, 95% CI 0.43, 1.10; *n* = 303, Figure 3; low certainty, Table S6).

Observational studies: Five studies (one case-control [76], three cohort [44,59,64] and one non-randomised arm of a randomised trial [36]) reported BPD for this comparison (Table 1). All were included in a meta-analysis that did not detect a difference in BPD (RR 1.01, 95% CI 0.72, 1.43, *I*^2^ = 39%, *n* = 1644, Figure 3, very low certainty, Table S6).

Overall: There is inconclusive evidence for an effect of pasteurisation of HM on BPD (Table 2).

### 3.5. Retinopathy of Prematurity (ROP)

ROP was reported in 29 studies and severe ROP in 17 [23,33,35,42,51,55,56,58,64,65,66,68,69,71,73,80,81] (Table 1). The International Classification of Retinopathy of Prematurity [84] was used to define ROP in most studies, with five studies providing no definition [21,22,34,37,51]. The definition of severe ROP varied and is detailed in Table 1.

#### 3.5.1. Comparison 1: Exclusive Human Milk vs. Exclusive Preterm Formula

**Figure 4:**
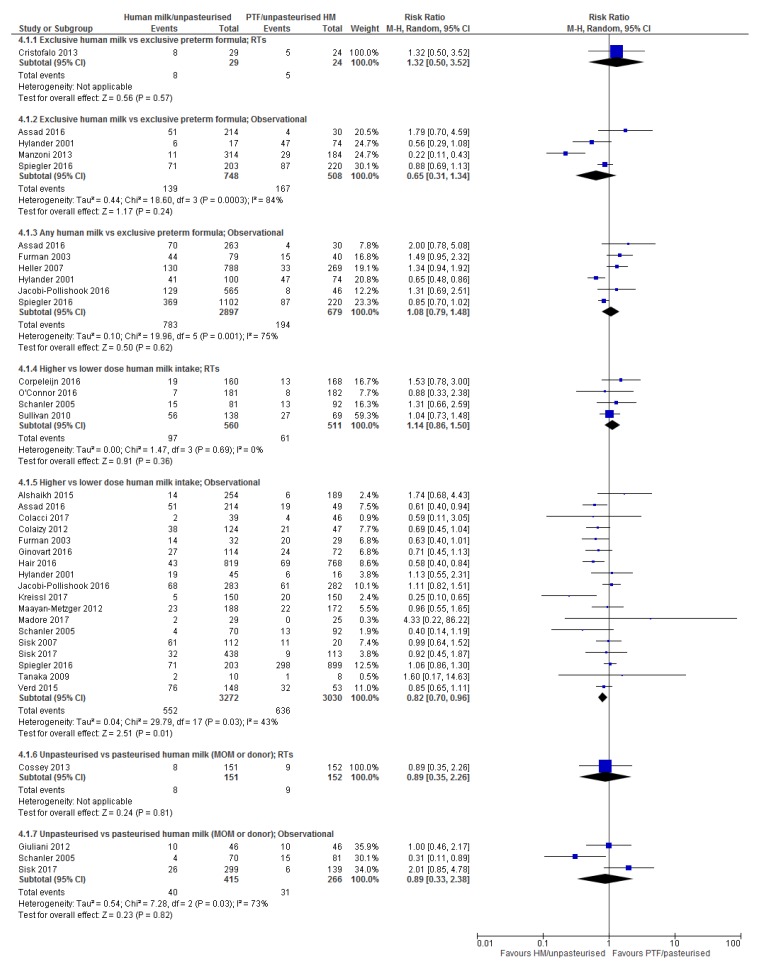
Forest plot of relative risk for the association between human milk and retinopathy of prematurity.

Randomised trials: One small RT [34] reported the effect of EHM compared with EPTF and did not detect a difference in ROP (RR 1.32, 95% CI 0.50, 3.52, *n* = 53, Figure 4; low certainty, Table S7).

No RTs reported severe ROP for this comparison.

Observational studies: Four studies (one interrupted time series [21] and three cohort [53,58,66], Table 1) reported the association between EHM and EPTF feeding on any ROP. No difference was detected in any ROP with this comparison (RR 0.65, 95% CI 0.31, 1.34; *n* = 1256, *I*^2^ = 84%, Figure 4; very low certainty, Table S7). The source of the substantial heterogeneity is unclear and likely due to a combination of differences in study design, baseline differences in the population and an imbalance of numbers in each group (Table 1).

Severe ROP was reported in three of the above studies [53,58,66] and on meta-analysis showed a reduction in severe ROP with an EHM diet (RR 0.23, 95% CI 0.07, 0.73; *n* = 1012, *I*^2^ = 57%, Figure S3; low certainty, Table S8).

Overall: The evidence for an effect of EHM compared with EPTF on ROP is inconclusive. There is a possible reduction in severe ROP with EHM (ARR 7.6%, from 2.7 to 9.1 fewer cases/100; Table 2).

#### 3.5.2. Comparison 2: Any Human Milk Compared with Exclusive Preterm Formula

Randomised trials: No RTs reporting ROP were identified for this comparison

Observational studies: Six observational studies including one interrupted time series [21] and five cohort studies [47,50,53,54,66] (Table 1) compared any HM with EPTF. No effect of feeding type on ROP was detected (RR 1.08, 95% CI 0.79, 1.48; *n* = 3576, *I*^2^ = 75%; Figure 4, very low certainty, Table S7). Overall, there was an imbalance of infants in groups (2897 and 679 in any HM and EPTF groups, respectively). Heterogeneity is likely due to the variation in HM intake (Table 1), and to the larger more mature infants in the EPTF group in three of the studies [21,47,56].

Three of these studies [50,53,66] reported severe ROP with similar findings (RR 0.81, 95% CI 0.42, 1.56; *n* = 2553, *I*^2^ = 74%, Figure S3; very low certainty, Table S8).

Overall: There is inconclusive evidence for an effect of any HM vs. EPTF on either ROP or severe ROP (Table 2).

#### 3.5.3. Comparison 3: Higher vs. Lower Dose Human Milk Intake

Randomised trials: Four RTs [32,35,36,37] were identified that compared higher vs. lower dose HM intake on the incidence of any ROP (Table 1). On meta-analysis, no difference in ROP was detected for this comparison (RR 1.14, 95% CI 0.86, 1.50; *n* = 1071, *I*^2^ = 0%, Figure 4; moderate certainty, Table S7). Two of these trials [35,36] also reported severe ROP and did not detect a difference (RR 1.15 95% CI 0.66, 2.02; *n* = 536, *I*^2^ = 0%, Figure S3; low certainty, Table S8). In addition to reporting severe ROP, Schanler et al. [36] reported the highest median stage of any ROP according to feeding group which was stage 1 for EHM groups (MOM and PDM) compared to stage 2 in the group supplemented with PTF, *p* = 0.04.

Observational studies: Nineteen observational studies, comprised of four interrupted time series [21,69,71,73], thirteen cohort [22,23,42,43,47,49,53,54,55,56,64,65,66,81], and one non-randomised arm of an RT [36], investigated the dose effect of HM on any ROP. Eighteen of these studies could be included in a meta-analysis and, contrary to the meta-analysis of RTs, showed a reduction in ROP (RR 0.82, 95% CI 0.70, 0.96; *n* = 6302, *I*^2^ = 43%, Figure 4; very low certainty, Table S7). Heterogeneity is likely due to the varying amounts of HM consumed in the higher vs. lower dose groups (Table 1), and that six studies [36,42,56,69,71,73] reported only severe ROP. Two studies [22,56] had particularly wide CIs.

Thirteen of these studies [23,36,42,49,53,55,56,64,65,66,69,71,73] reported severe ROP (Table 1) and were included in the meta-analysis. There was a significant reduction in severe ROP associated with higher dose HM (RR 0.63, 95% CI 0.46, 0.87; *n* = 5224; *I*^2^ = 22%, Figure S3; low certainty, Table S8).

One retrospective case control study [81] reported any ROP and feeding and could not be included in the meta-analysis. Porcelli et al. [81] found that HM intake in Postnatal Week 2 was an independent predictor for ROP surgery (OR = 0.94, CI not reported).

Overall: The evidence regarding high vs. low dose of HM on both ROP and severe ROP is inconclusive (Table 2).

#### 3.5.4. Comparison 4: Unpasteurised vs. Pasteurised Human Milk

Randomised trials: One RT [33], reporting severe ROP only (Table 1), did not detect a difference when unpasteurised HM was compared with pasteurised HM (RR 0.89 95% CI 0.35, 2.26, *n* = 303, Figure 4; low certainty, Table S7).

Observational studies: Three observational studies (one prospective case-control [76], one cohort study [64] and one non randomised arm of RT [36]) compared the effects of pasteurisation on any ROP (Table 1). Similar to the RT, there were no differences between feeding groups (RR 0.89, 95% CI 0.33, 2.38, *n* = 681, *I*^2^ = 73%, Figure 4, very low certainty, Table S7). Meta-analysis of the two studies reporting severe ROP [36,64] also did not detect a difference (RR 0.81 95% CI 0.13, 5.08, *n* = 589 infants, *I*^2^ = 86%, Figure S3; very low certainty, Table S8). The source of heterogeneity may be from differences in study design, and the variation in the relative dose of pasteurised and unpasteurised HM used (Table 1).

Overall: The evidence for an effect of pasteurisation of HM on any or severe ROP is inconclusive (Table 2).

### 3.6. Neurodevelopment

Neurodevelopment was reported in 13 studies (with 14 reports) comprised of one RT [35] and twelve cohort studies (13 reports) [22,39,40,42,46,48,54,56,57,60,62,67,68] (Table 1). There was variation between studies in the tools used to assess the outcome measures, with most studies using the Bayley Scale of Infant Development (BSID) second [40,42,46,48,54,56,57,60,68,81] or third edition [35,42,56] (Table 1). Other tests included the Alberta Infant Motor Scale (AIMS) [40] and the Kaufman Assessment Battery for Children (KABC) [22] (Table 1). As BSID II and III and KABC are standardized (mean 100, standard deviation 15), studies using these assessments have been pooled for meta-analyses. Other tests have been reported narratively.

#### 3.6.1. Comparison 1: Exclusive Human Milk vs. Exclusive Preterm Formula

No RTs or observational studies were identified for this comparison

#### 3.6.2. Comparison 2: Any Human Milk Compared with Exclusive Preterm Formula

Randomised trials: No RTs were identified for this comparison

Observational studies: Five cohort studies [40,48,54,57,68] reported the impact of any HM vs. EPTF on neurodevelopment. All studies used BSID II to assess cognition at 12 [40], 18 [54,68], 20 [48] or 30 [57] months of age; four also reported BSID II motor development [48,54,57,68] (Table 1).

**Figure 5:**
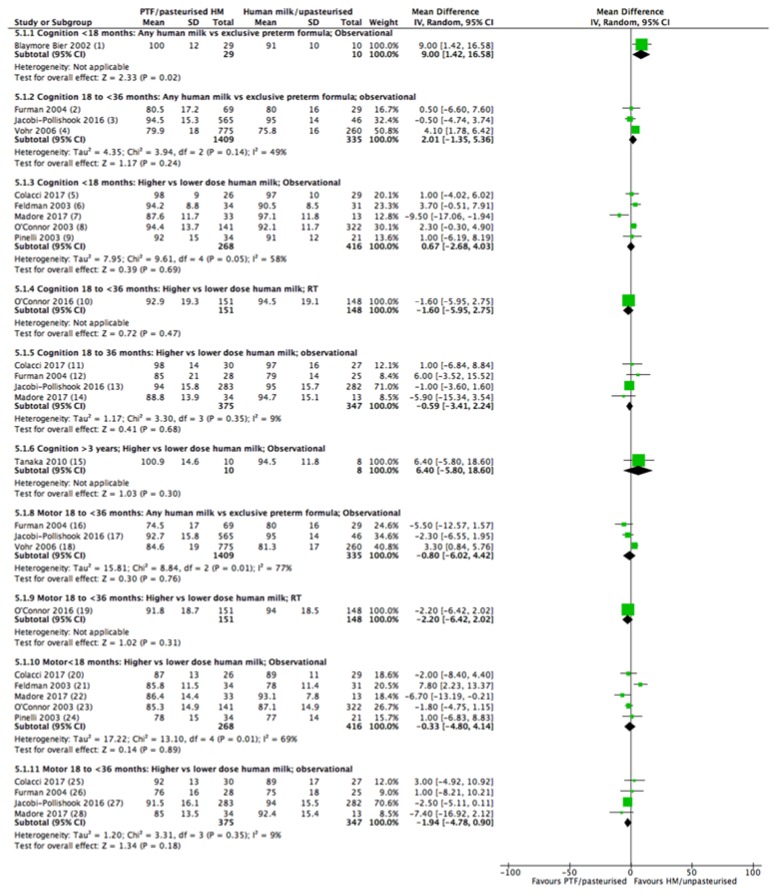
Forest plot of mean difference for association between human milk and neurodevelopmental scores. Footnotes: (1) BSID II MDI at 12 months, Mean adjusted for maternal Peabody Picture Vocabulary Test score and number days of oxygen; (2) BSID II MDI at 20 months; (3) BISD II MDI at 18 months; (4) BSID II MDI at 18 months; (5) BSID II MDI at 12 months CA; (6) BSID II MDI at 6 months CA; (7) BSID II MDI at 12 months; (8) BSID II MDI at 12 months CA; (9) BSID II MDI at 12 months; (10) BSID III MDI at 18 months CA, Adjusted Mean; (11) BSID III MDI at 18 months; (12) BSID II MDI at 20 months; (13) BSID II MDI at 18 months; (14) BSID III MDI at 2 years CA; (15) KABC five-year mental processing; (16) BSID II PDI at 20 months; (17) BSID II PDI at 18 months; (18) BSID II PDI at 18 months; (19) BSID III PDI at 18 months CA, Adjusted Mean; (20) BSID III PDI at 12 months CA; (21) BSID II PDI at 6 months CA; (22) BSID III PDI at one year CA; (23) BSID II PDI at 12 months CA; (24) BSID II PDI at 12 month; (25) BSID III PDI at 18 months; (26) BSID II at 20 months; (27) BSID II PDI at 18 months; (28) BSID III PDI at two years CA. Abbreviations: BSID, Bayley’s Scale of Infant Development; CA, corrected age; KABC, Kaufman Assessment Battery for Children; MDI, Mental Developmental Index; PDI, physical developmental scale.

Cognition: At <18 months of age cognitive development was reported in only one study for this comparison [40]. A significant increase in the mental development index (MDI) was found (mean difference (MD) 9 points, 95% CI 1.42, 16.58, *n* = 39, Figure 5; very low certainty, Table S9). In the age range 18 to <36 months, three studies were included in the meta-analysis [48,54,68] and did not detect a difference in MDI (MD 2.01 points, 95% CI -1.35, 5.36, *n* = 1744, *I*^2^ = 49%, Figure 5; very low certainty, Table S9).

Motor: One study showed better motor development at 12 months of age [40] (assessed using AIMS) in the human milk group vs. the formula group (63 ± 20% vs. 46 ± 15%, respectively, *n* = 39, *p* < 0.05). Three studies [48,54,68] could be included in the meta-analysis for the age range 18 to <36 months, with no difference detected in psychomotor development index (PDI) between feeding groups (MD −0.8 points 95% CI −6.02, 4.42, *n* = 1744, *I*^2^ = 77%, Figure 5; very low certainty, Table S9). Heterogeneity may be explained by the different population with 2 studies examining infants born in the late 1990s [48,68] and one using a cohort of infants born in 2005 [54], as well as differences in the dosage of HM.

The study by Vohr et al. (2007) [57] could not be included in the meta-analysis and showed that both Bayley MDI and PDI in the three highest quintiles of HM intake were significantly higher than the no HM group, *p* < 0.05 (mean MDI in no HM, 40th–60th, 60th–80th and >80th groups 76.5, 82.7, 86.4, 89.7 and mean PDI 78.4, 85.2, 87.3, 90.2 respectively) at 30 months corrected age (CA).

Overall: The evidence is inconclusive for an effect of any HM vs. EPTF on either cognitive or motor development (Table 2).

#### 3.6.3. Comparison 3: Higher vs. Lower Dose Human Milk Intake

Randomised trials: One RT [35] assessed the dose of HM on neurodevelopment, using adjusted means for BSID III MDI and PDI at 18 months corrected age (Table 1). No difference between feeding groups were found (MD −1.6, 95% CI −5.95, 2.75; −2.2 95% CI −6.42, 2.02 for cognition and motor scores, respectively, *n* = 299), Figure 5 moderate certainty, Table S9.

Observational studies: Ten studies reported a dose comparison effect on neurodevelopment and of these, eight cohort studies could be included in a meta-analysis [22,42,46,48,54,56,60,62] (Table 1).

Cognition: Five studies [42,46,56,60,62] reported this outcome for the age group <18 months and found no difference (MD 0.67, 95% CI −2.68, 4.03, *n* = 684, *I*^2^ = 58%), Figure 5, very low certainty, Table S9). Heterogeneity is likely due to differences in the dose of HM in the high and low groups (Table 1), and the time periods that the infants were born. Four studies [42,48,54,56] reported cognitive development for the age group 18 to <36 months and similarly found no effect of feeding type (MD −0.59, 95% CI −3.41, 2.24, *n* = 722, *I*^2^ = 9%, Figure 5, very low certainty, Table S9). One study reported no difference in cognitive development at over three years of age [22] (MD 6.4, 95% CI -5.8, 18.6, *n* = 18) Figure 5. 

Motor: Five studies (the same studies that reported cognition) [42,46,56,60,62] also reported motor development and found no difference in motor scores in the age group <18 months (MD −0.33, 95% CI −4.8, 4.14, *n* = 684, *I*^2^ = 69%, Figure 5). The same reasons for heterogeneity apply. Similarly, four studies [42,48,54,56] in the age group 18 to <36 months also found no difference (−1.94, 95% CI −4.78, 0.90, *n* = 722, *I*^2^ 9%, Figure 5). For both cognitive and motor development there is very little confidence that there is no effect of feeding type (Table S9).

Three additional studies could not be included in the meta-analysis but reported on this comparison. Belfort and co-workers’ cohort study [39] found that IQ was positively associated with the number of days that the infant received >50% human milk feeds (0.5 points/day, 95% CI 0.2, 0.8). Were and Bwibo [67] assessed a cohort of 120 preterm infants in Kenya and found an association between the use of EHM in the first month of life and functional disability at two years of age (RR 2.04, 95% CI 1.1, 3.78) *p* = 0.02). Vohr et al. (2007) [57] reported, for every 10 mL/kg/day increase in HM, at 30 months, the MDI increased by an estimated 0.59 points, *p* = 0.0005 and the PDI by 0.56 points, *p* = 0.009.

Overall: The evidence for an effect of high vs. low dose HM on both cognitive and motor development is inconclusive.

#### 3.6.4. Comparison 4: Unpasteurised vs. Pasteurised Human Milk

No studies were identified for this comparison.

## 4. Discussion

### 4.1. Summary of Main Results

Six RTs with 1472 infants and 43 observational studies with 14,950 infants were included in this systematic review. Both EHM and any HM, compared with EPTF, reduced NEC. A higher proportion of HM was more effective than lower amounts with a 4% ARR in any NEC and 2% reduction in severe NEC. This supports a policy of moving to 100% human milk for NEC protection when mothers are unable to meet all their infant requirements. An EHM diet was associated with a possible 5% reduction in LOS, however there does not appear to be a dose effect. There is inconclusive evidence for an effect of exclusive or any HM on the incidence of BPD or ROP, except for a possible effect of EHM, compared with formula, on reduction of severe ROP with a 7.6% reduction. We also found insufficient evidence to draw any conclusions regarding the role of HM on neurodevelopment. This outcome was complicated by the variation in the timing of testing, and the different tests used. What is clear is that the mean differences between feeding groups is small and hence large numbers will be required to show an effect. Many individual studies included in this meta-analysis are not sufficiently powered to determine these differences. The overall evidence for the effect of pasteurisation was inconclusive except for possibly no effect on LOS.

### 4.2. Strengths and Limitations

In this review, we have used robust methods to search, synthesise and critique evidence on this topic. We have combined five major morbidities on preterm infants into the one review, providing a comprehensive overview that is relevant to neonatal clinicians and will inform clinical decisions regarding feeding, particularly of DHM. In addition, we have attempted to differentiate the effects of various combinations of HM and PTF by synthesising data in four distinct comparisons, each designed to answer a particular question.

It was beyond the scope of the review to determine the effect of introducing a bovine derived, compared to a human derived, fortifier. We also limited our search to English language which may have failed to retrieve some literature. 

For each meta-analysis, we used standard Cochrane methods for presenting pooled results—these methods appropriately give greatest weight to large studies and/or large number of events. For example, in the NEC meta-analysis (Figure 1), two large studies [66,68] provided most of the data, and thus the greatest weight, showing a clear advantage with use of any human milk compared with exclusive preterm formula. 

While our inclusion criteria stipulated our population and outcomes of interest, we still encountered heterogeneity with some studies choosing to study only very preterm infants (<1000 g or <1250 g) which were a more vulnerable subset of our population of interest and may limit applicability. The majority of studies included in the meta-analysis were from developed countries, reflecting modern NICU practice, making these results quite generalisable. A large source of variability in the studies arose from the exposure to HM. Most studies measured exposure over the neonatal admission whereas some focussed on early feeding only. There was considerable heterogeneity in the dose of HM within each group and this was particularly so in the “any HM vs. EPTF” and the “high vs. low dose” HM groups which could vary from as high as EHM to the lower 20% of intake, or was not measured at all in many cases. Where heterogeneity was substantial, the certainty of the evidence was downgraded to reflect this, and so for many of the outcomes we are uncertain about the evidence despite quite large numbers of studies included in the meta-analyses. The true effect may be substantially different from the estimate provided from these studies and more studies of robust design are needed to increase our confidence. In addition, the fortifier used for HM was generally bovine derived but sometimes human derived and we did not differentiate between these as this was beyond the scope of this review. Nevertheless, the avoidance of bovine protein in an otherwise EHM diet, may have an impact which we have failed to take into account. Finally, another source of heterogeneity arises from the various definitions of the outcomes used, and in the case of neurodevelopment, the tools used to measure this.

All six RTs were assessed as low risk of bias and the observational studies varied with 26 considered low risk, 14 as moderate and 3 as high risk of bias. Our risk of bias assessment did not take into account poor statistical methods, typical of many of the observational studies, as this is not relevant to a meta-analysis, but makes individual study results unreliable. Additionally, many of the studies had a small sample size or were designed to answer a different question and included the outcomes of interest as secondary outcomes, hence were often not powered to detect small differences.

### 4.3. Findings from Other Reviews

Two recent narrative systematic reviews [85,86] and three meta-analyses [87,88,89] have been published on this topic. Cacho et al. [85] reviewed the evidence for the effect of DHM, EHM and the dose of HM, on NEC and, in line with our results, showed no clear evidence that DHM compared with formula reduces NEC, while an EHM diet may be protective and a higher dose of HM reduces the risk of NEC.

De Silva et al. [87] conducted a narrative review of infection rates in preterm infants. Of the nine studies they included, five were not included in our review due to being published prior to 1990 (*n* = 3), the study population not meeting our inclusion criteria (*n* = 1) or not published in English (*n* = 1). De Silva et al. concluded that the literature overall did not support a benefit of HM in preventing LOS, despite some small studies showing a protective effect and poor study design in many of the included studies. Our review included a larger number of more recent studies and despite this only found possible evidence of a protective effect EHM vs. EPTF. A recent meta-analysis of the effect of DHM (+/− MOM) vs. PTF on BPD by Villamor-Martinez [88] with considerable overlap of studies in our review, found no effect from the seven RTs included in their review but eight observational studies showed reduced BPD with DHM. However, our certainty of this finding, as determined by GRADE, is very low; hence we have given more weight to the RT results. A recent meta-analysis of observational studies on the effect of HM on ROP by Zhou et al. [89] used comparisons which overlapped with ours, and showed a protective effect of HM on ROP and severe ROP for both EHM vs. EPTF and “mainly HM vs. mainly formula” which equates to our high vs. low dose HM group. Similar to our findings, Zhou et al. found no effect in the “any HM vs. EPTF” group. In a narrative review of neurodevelopment, which included many of the same studies as in our review, Lechner and Vohr [85] presented evidence of a small protective effect of HM but also acknowledge the challenges of studying an outcome that has so many confounding variables such as parental IQ and associated socioeconomic differences. They also highlighted the lack of high quality studies in this area and the need to control for confounding variables.

In addition, one study [90], which we were unable to include because the outcomes were reported as a composite, also found an association between HM (during the first 10 days of life) and improved outcomes. In their retrospective review of 349 infants born weighing <1500, any HM in the first five days of life was associated with a lower incidence of NEC, LOS and/or death. During Days 6–10, it was only when HM intakes were >50% of the total intake was a protective effect elicited.

## 5. Conclusions

### 5.1. Implications for Practice

We have shown evidence of a clear protective effect of HM against NEC and a possible reduction in LOS, severe ROP and severe NEC. In addition, we have shown that any HM is better than none, that the more HM the preterm infant receives the better the outcome, and that for NEC there is an advantage in topping up infants who are already receiving quite large proportions of their enteral intake as HM, to EHM. From a clinical perspective, it would seem just as important to offer DHM to an infant who is getting nearly all MOM as it is for an infant who is getting none.

### 5.2. Implications for Research

The benefits of HM feeding are difficult to study given that it is not ethical to randomise breast feeding. However, there is a need for large and well conducted studies, designed to answer specific questions, particularly in relation to the effects of DHM and pasteurisation.

## Figures and Tables

**Table 1:** Characteristics of included studies.

Study, Country	Design	Participants	Intervention, [Proportion of HM % Unless Stated Elsewhere]	Comparisons for This Review C1: EHM vs. EPTF C2: Any HM vs. EPTF C3: High vs. Low Dose HM C4: Unpasteurised vs. Pasteurised	Study Duration	Outcomes	Risk of Bias
		BW, g; GA, Wk; *n*					
**RANDOMISED TRIALS**							
Corpeleijn (2016) [32] Netherlands	RT	373 infants Gp1: 1065 (830, 1265); 28.3 ± 2.3; 183 Gp2: 1077 (854, 1275); 28.6 ± 2.2; 190	Gp1: MOM + PDHM [100%] Gp2: MOM + PTF [median 84.5%]	C3: Gp1 vs. Gp2	Intervention 1st 10 d of life Outcomes measured through hospital duration	NEC (≥Bell’s stage 2) Severe NEC (requiring surgery) Sepsis ((+)ve blood culture) BPD (need for O_2_ for ≥28 d) ROP (all stages)	Low
Cossey (2013) [33] Belgium	RT	303 infants Gp1: 1291 ± 353; 30 (28, 31); 151 Gp2: 1,270 ± 406; 30 (28, 31); 152	Gp1: Unpasteurised MOM [86% (61, 95) Gp2: Pasteurised MOM [88% (54, 95)] PTF used in both gps if MOM insufficient	C3: (sepsis only) per 10 mL/kg increase in MOM C4: Gp1 vs. Gp2	SS: Birth SE: 8 wk of life or discharge	NEC (≥Bell’s stage 2) Severe NEC (requiring surgery) Sepsis ((+)ve blood culture + clinical signs for >48 h) BPD (O_2_ @ 36 wk) Severe ROP (requiring surgery)	Low
Cristofalo (2013) [34] USA and Austria	RT	53 infants Gp1: 996 ± 152; 27.7 ± 1.5; 29 Gp2: 983 ± 207; 27.5 ± 2.4; 24	Gp1: EHM (HMDF), [100%] Gp2: EPTF (BovF), [0%]	C1: Gp1 vs. Gp2	SS: Start of enteral nutrition SE: Earliest of 91 d of age, DC, 50% of oral feeds	NEC (≥Bell’s stage 2) Severe NEC (requiring surgery) Sepsis ((+)ve blood culture + clinical signs for >5 d) ROP (not defined)	Low
O’Connor (2016) [35] Canada	RT	363 infants Gp1: 995 ± 273; 27.5 ± 2.4; 181 Gp2: 996 ± 272; 27.8 ± 2.7; 182	Gp1: EHM (MOM + DHM) [100%; MOM 58.4% (13.6, 96)] Gp2: Mixed feeding (MOM + PTF) [MOM 63.3% (9.6, 97.2)]	C3: Gp1 vs. Gp2	SS: d of consent (within 96 h of birth) SE: after 90 d	NEC (≥Bell’s stage 2) Sepsis, ((+)ve blood or CSF culture) BPD (O_2_ @ 36 wk) Severe ROP (stage 4/5, laser/intraocular injection) Neurodevelopment (BSID)	Low
Schanler (2005) [36] USA	RT	243 infants Gp1: 947 ± 233; 27 ± 2; 81 Gp2: 957 ± 267; 27 ± 2; 92 Gp3: 999 ± 259; 27 ± 2; 70	Gp 1: PDM as supplement to HM (100%) Gp 2: PTF as supplement to HM [NR] Gp 3: EHM (reference, non-randomised) [100%]	C3: Gp1 vs. Gp2 (RT) and Gp3 vs. Gp2 (observational) C4: Gp3 vs. Gp1 (observational)	SS: Enrolment (4 d) SE: 90 d of age or DC	NEC (≥Bell’s stage 2) Sepsis ((+)ve blood culture + clinical signs for >5 d) BPD (O_2_ @ 36 wk) ROP (all stages)	Low
Sullivan (2010) [37] USA and Austria	RT	207 infants Gp1: 945 ± 202; 27.2 ± 2.2; 67 Gp2: 909 ± 193; 27.1± ± 2.3; 71 Gp3: 922 ± 197; 27.3 ± 2.0; 69	Gp 1: EHM + HMDF (fortified at 100 mL/kg) [100%; (MOM 73% (16, 82)] Gp 2: EHM + HMDF (fortified at 40 mL/kg) [100%; (MOM 70 (18, 80)] Gp 3: Mixed + BovF [82% (38, 100)]	C3: Gps1,2 vs. Gp3	SS: Start of enteral nutrition SE: Earliest of 91 d of age, DC, 50% oral feedings	NEC, (clinical + radiographic evidence) and requiring surgery Sepsis (clinical signs) BPD (O_2_ @ 36 wk) ROP (not defined)	Low
**OBSERVATIONAL STUDIES**							
Alshaikh (2015) [69] Canada	Interrupted time series	443 infants Gp1: 1236 ± 390; 28.5 ± 2.3; 189 Gp2: 1186 ± 373; 28.5 ± 2.1; 254	Gp1: Pre-quality improvement [NR] Gp2: Post- quality improvement [NR] Strategies designed to improve intake of MOM	C3: Gp2 vs. Gp1	Not reported	NEC (≥Bell’s stage 2) Sepsis ((+)ve blood or CSF culture) BPD (O_2_ @ 36 wk) Severe ROP (not defined)	Low
Assad (2016) [21] USA	Interrupted time series	293 infants BW: Range: 490 to 1700 GA: Gp 1: 27.7 ± 2.7; 87 Gp 2: 28.3 ± 2.8; 127 Gp 3: 27.6 ± 2.8; 49 Gp 4: 29.8 ± 2.5; 30	Gp1: Human – EHM + HMDF [100 (MOM + DHM)] Gp 2: Bovine – EHM + BovF [100 (MOM + DHM)] Gp 3: Mixed—not further defined [NR] Gp 4: EPTF—not further defined [0%]	C1: Gp 1,2 vs. Gp 4 C2: Gp 1,2,3 vs. Gp 4 C3: Gp 1,2 vs. Gp 3 NEC: C3: Gp1 vs. Gp 2,3	Not reported	NEC (≥Bell’s stage 2) (data only presented for C3) Sepsis (not defined) BPD (O_2_ @ 36 wk) ROP (not defined)	Low
Belfort (2016) [39] Australia	Cohort	180 infants Whole cohort 947 ± 210; 27.3 ± 1.8	No. of d infants received >50% enteral intake as HM until d 28	C3: synthesised narratively	Intervention first 28 d of life Ax at 7 years	Neurodevelopment (BSID)	Moderate (recruitment unclear and some loss to FU)
Bensouda (2013) [74] Canada	Retrospective case-control study	114 infants Cases: 1069 (845, 1320); 27.2 (26, 30); 38 Controls: 1058 (877, 1268); 27.8 (27, 30); 76	Gp 1: Mixed (NEC), 38 Gp 2: Mixed (No NEC), 76	C3: synthesised narratively	Not reported	NEC (≥Bell’s stage 2)	Low
Bishop (2010) [70] USA	Interrupted time series	331 infants Gp1: 1056 ± 279; 28.5 ± 2.7; 179 Gp2: 1059 ± 289; 28.6 ± 2.9; 152	Gp 1: Pre-PDM era [HM 51%, PTF ~49%] Gp 2: Post-PDM era [HM 75%, PTF ~25%]	C3: Gp 2 vs. Gp1	Unclear, feeding data collected from birth to 34 wk CA	NEC (≥Bell’s stage 2) Severe NEC (requiring surgery) Sepsis ((+)ve blood or 2 CSF culture) BPD (O_2_ @ 36 wk)	Low
Blaymore-Bier (2002) [40] USA	Cohort	39 infants Gp1: 1174 ± 305; 28.6 ± 2.9; 29 Gp2: 1198 ± 170; 29.9 ± 2.2; 10	Gp1: Mixed feeding [878 (77, 1754) mL/wk of HM and 43 (0, 1051) mL/kg/wk of PTF] Gp2: EPTF [0%]	C2: Gp1 vs. Gp2	Duration of hospitalisation	NEC (not defined) Sepsis (not defined) Neurodevelopment (BSID)	Moderate (convenience sample)
Chowning, (2016) [41] USA	Cohort	550 infants Whole cohort: 1.05 ± 0.28 kg; 28.4 ± 2.6 *n* = Gp1: 260; Gp2, 290; Gp 3, 76; Gp4, 71	Gp1: <50% d received HM Gp2: ≥50% d received HM Separate analysis done for extremes of intake: Gp3: 0% d received HM, Gp4: ≥50% d received HM	C2: Gp4 vs. Gp3 C3: Gp2 vs. Gp1	Duration of hospitalisation	NEC (≥Bell’s stage 2) Severe NEC (requiring surgery)	Low
Colacci (2017) [42] USA	Cohort	85 infants Gp1: 783 ± 143; 26 ± 1.9; 39 Gp2: 770 ± 137; 26 ± 1.9; 46	Gp1: EHM + HMDF for first 4 wk of life [100%; MOM used for any feedings 92% of times] Gp2: Mixed feeding + BovF [83 (17, 100) % of feedings as formula]	C3: Gp1 vs. Gp2	First 4 wk of life	NEC (≥Bell’s stage 2) Sepsis ((+)ve blood culture) BPD (O_2_ @ 36 wk) Severe ROP (requiring treatment) Neurodevelopment (BSID)	Low
Colaizy (2012) [43] USA	Cohort	171 infants Whole cohort: 889 (724, 1064); 27 (25.4, 28.9) *n* = Gp1, 17; Gp2, 30, Gp3, 36, Gp4, 88	Gp 1: <25% HM, Gp 2: 25–50% HM Gp 3: 50–75% HM Gp 4: >75% HM Combined MOM and PDM and PTF	C3: Gps 3,4 vs. Gps 1,2	SS: Initiation of enteral feedings SE: discharge	NEC (≥Bell’s stage 2) Sepsis ((+)ve blood culture + clinical signs for >3 d) BPD (DC on O_2_) ROP (all stages)	Low
Dicky (2017) [44] France	Cohort	63 NICUs, 926 infants Gp1: 1285 ± 348; 29.1 ± 1.9; 33 NICUs, 290 Gp2: 1267 ± 338; 29.1 ± 1.9; 30 NICUS, 636	Gp1: NICUs who pasteurised MOM until 33 wk PMA, [NR] Gp2: NICUs who did not pasteurise MOM, [NR] Infant diet may also be supplemented with pasteurised donor milk or formula (un-measured)	C4: Gp 2 vs. Gp1	Duration of hospital admission	NEC (Bell’s stge2/3) Sepsis ((+)ve blood culture + clinical signs for >3 d) BPD (O_2_ @ 36 wk ± need for O_2_ for ≥28 d)	Low
Feldman (2003) [46] Eidelman (2004) [45] Israel	Cohort	86 infants Whole cohort: 1298 ± 335.6; 30.4 ± 3 *n* = Gp1, 34; Gp2, 21; Gp3, 31	Gp 1: >75% HM (MOM) Gp 2: 25–75% HM (MOM) Gp 3: <25% HM (MOM)	C3: Gp1 vs. Gp3	Duration of hospitalisation	Neurodevelopment (BSID)	Low
Fonseca (2017) [75] Brazil	Retrospective case-control study	323 infants Gp1: 989 (495, 1795); 28.2 (22, 33); 94 Gp2: 1287 (750–950 *); 31 (25.4, 36.5); 229	Gp1: With BPD, 94 Bp2: Without BPD, 229	C3: synthesised narratively	SS: Birth SE: 42 d or discharge	BPD (need for O_2_ for ≥28 d)	Moderate (some differences between gps)
Furman (2003) [47], Furman (2004) [48] USA	Cohort	119 infants Gp1: 1103 ± 260; 28 ± 2; 40 Gp2: 914 ± 205; 26 ± 2; 29 Gp3: 988 ± 248; 27 ± 2; 18 Gp4: 1163 ± 225; 28 ± 2; 32	Gp 1: EPTF [0%] Gp 2: 1–24 mL/kg HM (MOM) Gp 3: 25–49 mL/kg HM (MOM) Gp 4: ≥50 mL/kg HM (MOM)	C2: Gps 2,3,4 vs. Gp 1 C3: Gp 4 vs. Gp 2	SS: Initiation of oral HM SE: wk 4 of life	NEC (≥Bell’s stage 2) Sepsis ((+)ve blood culture + clinical signs for >5 d) BPD (O_2_ @ 36 wk) ROP (all stages) Neurodevelopment (BSID)	Moderate (some differences between gps)
Ginovart (2016) [49] Spain	Cohort	186 infants Gp1: 1078 ± 289; 29.1 ± 2.9; 114 Gp2: 1108 ± 273; 29.6 ± 2.9; 72	Gp1: EHM (MOM + PDHM) [100%] Gp2: Any PTF (mixed feeding) [NR]	C3: Gp1 vs. Gp2	Duration of neonatal admission	NEC (not defined) and requiring surgery Sepsis (not defined) ROP (all stages)	Low
Giuliani (2012) [76] Italy	Prospective case-control study	92 infants Gp1:984 ± 230; 28.3 ± 2.3; 46 Gp2:968 ± 236; 28.3 ± 2.3; 46	Gp 1: >80% Unpasteurised MOM during first 20 d Gp 2: >80% PDM during first 20 d	C4: Gp1 vs. Gp2	Not reported	NEC (≥Bell’s stage 2) Severe NEC (requiring surgery) BPD (O_2_ @ 36 wk ± need for O_2_ for ≥28 d) ROP (all stages)	Low
Hair (2016) [71] USA	Interrupted time series	1587 infants Gp1: 844 ± 210; 26.5 ± 2.5, 819 Gp2: 823 ± 205; 26.4 ± 2.3, 768	Gp1: EHM (MOM + DHM) + HMDF [100%] Gp2: MOM + BovF + PTF [NR]	C3: Gp1 vs. Gp2	Varied: 60 d of age (2 sites) 34 wk PMA (2 sites)	NEC (not defined) Sepsis ((+)ve blood or CSF culture) BPD (O_2_ @ 36 wk) Severe ROP (Threshold ROP)	Moderate (some differences between gps)
Heller (2007) [50], Vohr (2006) [68], Vohr (2007) [57] USA	Cohort	1035 infants Gp1: 775 ± 134; 26.0 ± 2; 976 Gp2: 783 ± 140; 26.2 ± 2; 353 Neurodevelopment *n* = Gp1, 80; Gp2, 94; Gp3, 110; Gp4, 120; Gp5, 135; Gp6, 134	Gp 1: Any HM (MOM) [Median volume 30 mL/kg/d (6, 83) Gp 2: EPTF [0%] Also reported quintiles of intake for neurodevelopment Gp1: EPTF Gp2: <20th (23 mL/kg/d) Gp3: 20th–40th (≤53 mL/kg/d) Gp4: 40th to 60th (≤83 mL/kg/d) Gp5: 60th to 80th (≤112 mL/kg/d) Gp6: >80th (>112.5 mL/kg/d)	C2: Gp 1 vs. Gp 2 C2: Gps 2 to 6 vs. Gp1 C3: Gps 5,6 vs. Gps 2,3 Vohr 2007 synthesised narratively	Duration of neonatal admission with 18-month outcome follow up for neurodevelopment	NEC (not defined) Sepsis ((+)ve blood culture) BPD (O_2_ @ 36 wk) Severe ROP (requiring surgery) Neurodevelopment (BSID)	Low
Henderson (2009) [77] UK	Prospective case-control study	106 infants Gp1: 1114 ± 427; 27.9 ± 3.1; 53 Gp2: 1179 ± 478; 28.0 ± 2.7; 53	Gp1: NEC cases Gp2: Controls	C3: Synthesised narratively	Duration of neonatal admission	NEC (Bell’s all stages)	Low
Herrmann, (2014) [72] USA	Interrupted time series	548 infants Gp1: 1334 ± 436; 29.7 ± 2.5; 386 Gp2: 1.361 ± 542; 29.6 ± 3.0; 162	Gp1: Time 1: pre DHM and HMDF [NR] Gp2: Time 2: EHM incl HMDF [100%]	C3: Gp 2 vs. Gp1	SS: birth SE 33 wk PMA	NEC (≥Bell’s stage 2) Sepsis ((+)ve blood culture)	Low
Huston (2014) [51] USA	Cohort	361 infants Gp1: 919 ± 269; 26.7 ± 2.4; 44 Gp2: 1104 ± 262; 28.1 ± 2.2; 224 Gp3: 1177 ± 222; 29.1 ± 1.8; 93	Gp1: EHM (MOM or DHM + HMDF [100%] Gp2: DHM (MOM + DHM + BovF [100%] Gp3: PTF (EPTF or MOM+ BovF + PTF) [NR]	C3: Gps1,2 vs. Gp3	Not reported	NEC (≥Bell’s stage 2) Sepsis (not defined) ROP (not defined), Severe ROP (stage 3)	Moderate (some differences between gps)
Hylander (1998) [52], Hylander (2001) [53] USA	Cohort	212 infants Gp1: 1061 ± 251; 28.2 ± 2.3; 123 Gp2: 988 ± 242; 27.8 ± 2.4; 89 ROP: *n* = Gp1, 18; Gp2, 47; Gp3, 31; Gp4, 27; Gp5, 74	Gp 1: Any HM [NR] Gp 2: EPTF [0%] ROP reported as % HM Gp1: <20% Gp2: 20–79% Gp3: 80–99% Gp4: 100% Gp5: Formula	NEC, Sepsis, BPD C2: Gp1 vs. Gp2 ROP reported as C1: Gp 4 vs. Gp 5 C2: Gps 1,2,3,4 vs. Gp 5 C3: Gp 1 vs. Gp 3,4	Duration of neonatal admission	NEC (≥Bell’s stage 2) Sepsis ((+)ve blood culture + clinical signs) BPD (not defined) ROP (all stages)	Moderate (some differences between gps)
Jacobi-Polishook (2016) [54] Australia	Cohort	611 infants Median (range) Whole cohort 1350 (320, 262); 30 (25, 32) *n* = Gp1, 141; Gp2, 141; Gp3, 142; Gp4, 141; Gp5, 46	Gp1: 1st quartile of HM intake Gp2: 2nd quartile Gp3: 3rd quartile Gp4: 4th quartile Gp5: EPTF	C2: Gps1,2,3,4 vs. Gp5 C3: Gps 3,4 vs. Gps 1,2	Duration of neonatal admission	NEC (not defined) Sepsis (not defined) BPD (O_2_ @ 36 wk) ROP (all stages) Neurodevelopment (BSID)	Moderate (some differences between gps)
Johnson (2015) [78], Patel (2013) [82], Patel (2017) [83] USA	Prospective case-control study	*n* varies per study. Largest cohort = 291 infants Gp1:1050 ± 200; 27.2 ± 2.2; 29 Gp2:1065 ± 261; 28.2 ± 2.4; 262	HM exposure measured Gp1: NEC cases Gp2: non-NEC	C3: Synthesised narratively	Exposure (HM intake) varied: Johnson: 1st 14 d Patel 2013: 1st 28 d Patel 2017: birth to 36 wk or discharge	NEC (≥Bell’s stage 2) Sepsis ((+)ve blood culture) BPD (O_2_ @ 36 wk)	Moderate (some differences between gps)
Kimak (2015) [79] Brazil	Prospective case-control study	1028 infants Whole cohort: 1170 (900, 1340); 31 (29, 2) *n* = Gp1, 55; Gp2, 973	Gp1: NEC cases, 55 Gp2: controls, 973	C3: Synthesised narratively	SS: Birth SE: First of 30th d of life NEC or death	NEC (≥Bell’s stage 2)	Low
Kreissl (2017) [73] Austria	Interrupted time series	300 infants Median (range) Gp1: 1008 (450, 1490); 196 (163, 223) d; 150 Gp2: 896 (380,1480); 191 (163, 219) d; 150	Gp1: EHM (MOM + single PDHM [100%] Gp2: Mixed feeding (MOM + PTF) [NR]	C3: Gp1 vs. Gp2	Exposure: From start until full enteral feeds. Outcomes measured throughout neonatal admission	NEC (≥Bell’s stage 2) Sepsis (not defined) BPD (not defined) Severe ROP (≥stage 3)	Moderate (some differences between gps)
Maayan-Metzger (2012) [55] Israel	Cohort	360 infants Gp1: 1305 ± 388; 30.5 (28, 32); 188 Gp2: 1425 ± 399; 31 (29, 32); 172	Gp 1: HM ≥5 of 8 meals Gp 2: PTF ≥5 of 8 meals	C3: Gp1 vs. Gp2	SS: Birth SE: End of first month of life	NEC, (Clinical ± radiographic evidence) Sepsis ((+)ve blood culture + clinical signs for >3 d) BPD (need for O_2_ for ≥28 d) ROP (all stages) Severe ROP (stage 3)	Moderate (some differences between gps)
Madore (2017) [56] USA	Cohort	81 infants Gp1: 936.6 ± 211; 27 ± 1.5; 29 Gp2: 890.5 ± 175.8; 27.1 ± 1.9; 27 Gp3: 913.8 ± 222.6; 27.3 ± 2.1; 25	Gp1: only MOM over first m of life [100%] Gp2: >50% feeds as DHM over first m of life [100%] Gp3, >50% PTF over first m of life [>50% PTF]	C3: Gp1,2 vs. Gps3	SS: birth SE: after 1st month of life	NEC (≥Bell’s stage 2) Sepsis ((+)ve blood culture) BPD (O_2_ @ 36 wk) Severe ROP (requiring surgery) Neurodevelopment (BSID)	Low
Manea (2016) [38] Romania	Non randomised trial	34 infants Whole cohort: Range; 850 to 1000; 25 to 33 *n* = Gp1, 18, gp2, 16	Gp1: EHM [100%] Gp2: EPTF [0%]	C1: Gp1 vs. Gp2	Not reported	NEC (clinical signs) Sepsis (clinical signs)	High (group characteristics and participant flow not described)
Manzoni (2013) [58] Italy	Cohort	498 infants Gp1:1125 ± 247; 29.4 ± 2.5; 314 Gp2: 1100 ± 272; 29.2 ± 2.8; 184	Gp 1: EHM (MOM) [100%] Gp 2: EPTF [0%]	C1: Gp 1 vs. Gp	SS: Enrolment at <72 h of life SE: discharge or death	NEC (≥Bell’s stage 2) LOS ((+)ve blood culture + clinical signs) ROP (all stages) Severe ROP (threshold ROP)	Low
Montjaux-Régis (2011) [59] France	Cohort	48 infants Whole cohort: 1105 ± 282; 28.6 ± 1.5 *n* = Gp1, 20; Gp2, 11; Gp3, 17	Gp 1: <20% MOM Gp 2: ≥20% to <80% MOM Gp 3: ≥80% MOM	C4: Gp3 vs. Gp1	SS: Full enteral feeding with HM SE: weight 1400 g ± 32 wk GA	NEC (≥Bell’s stage 2) Sepsis (nosocomial infection) BPD (O_2_ @ 36 wk)	Low
O’Connor (2003) [60] UK and USA	Cohort	463 infants Gp1: 1275 ± 312; 29.7 ± 2.0; 43 Gp2: 1287 ± 279; 29.6 ± 1.9; 98 Gp3: 1288 ± 287; 29.5 ± 2.1; 203 Gp4: 1332 ± 279; 29.9 ± 2.0; 119	Gp 1: >80% HM at term CA + <100 mL/kg BW of PTF for duration of stay Gp 2: ≥50% energy from HM Gp 3: <50% energy from HM Gp 4: >80% PTF at term corrected age + <100 mL/kg BW of HM for duration of stay	C3: Gps 1,2 vs. Gps 3, 4	SS: Initiation of enteral feeding SE: Term CA (HM feeding exposure) or hospital discharge (NEC outcome)	NEC (not defined) Sepsis (not defined) BPD (O_2_ @ 36 wk) Neurodevelopment (BSID)	Moderate (some differences between gps)
Okamoto (2007) [80] Japan	Retrospective case-control study	14 infants. Median (range) Gp1:660 (504-812); 24 (23-26); 7 Gp2: 736 (660-804); 24 (24-25); 7	Gp 1: Mixed (Retinal detachment) Gp 2: Mixed (Non-retinal detachment)	C3: synthesised narratively	Not reported	Severe ROP (retinal detachment)	Low
Parker (2012) [61] USA	Cohort	80 infants Whole cohort: 1044 ± 246.5; 27.8 ± 2.1 *n* = Gp1, 40; Gp2, 40	Gp 1: Minimum 50% feed volume HM [≥50%] Gp 2: EPTF [0%]	C2: Gp 1 vs. Gp 2	Duration of neonatal admission	NEC (clinical ± radiographic evidence) Sepsis ((+)ve blood culture)	Low
Pinelli (2003) [62] Canada	Cohort	148 infants, 137 at 12m Ax Gp1: 1130 ± 244; 29 ± 3; 67 Gp2: 1090 ± 273; 29 ± 3; 70	Gp 1: >80% HM (MOM) Gp 2: <80% HM (MOM) or no HM	C3: Gp1 vs. Gp2	SS: during neonatal admission	Neurodevelopment (BSID)	Moderate (some loss to FU)
Porcelli (2010) [81] USA	Retrospective case-control study	77 infants Gp1: 873 ± 85; 26.4 ± 1.3; 66 Gp2: 842 ± 78; 25.9 ± 0.9; 11	Gp 1: Mixed (No ROP surgery) Gp 2: Mixed (ROP Surgery)	C3: synthesised narratively	Duration of neonatal admission	ROP (any grade) Severe ROP (requiring surgery)	Low
Schanler (1999) [63] USA	Cohort	108 infants Gp1: 1069 ± 169; 27.9 ± 1.2; 62 Gp2: 1044 ± 185; 27.9 ± 1.1; 46	Gp 1: Minimum 50 mL/kg/d of any HM [96 ±23 mL/kg/d] Gp 2: EPTF (0)	C2: Gp 1 vs. Gp2	Duration of neonatal admission	NEC (clinical ± radiographic evidence) Severe NEC (requiring surgery) Sepsis ((+)ve blood culture + clinical signs for >5 d)	Low
Schanler (2005) [36] USA	RT with 1 non-randomised arm	243 infants Gp1: 947 ± 233; 27 ± 2; 81 Gp2: 957 ± 267; 27 ± 2; 92 Gp3: 999 ± 259; 27 ± 2; 70	Gp 1: PDM as supplement to HM [100%] Gp 2: PTF as supplement to HM [NR] Gp 3: EHM (reference, non-randomised) [100%]	C3: Gp1 vs. Gp2 (RT) and Gp3 vs. Gp2 (observational) C4: Gp3 vs. Gp1 (observational)	SS: Enrolment (4 d) SE: 90 d of age or DC	NEC (≥Bell’s stage 2) Sepsis ((+)ve blood culture + clinical signs for >5 d) BPD (O_2_ @ 36 wk) ROP (all stages)	Low
Sisk (2007) [65] USA	Cohort	202 infants Gp1: 1112.8 ± 17.8; 28.1 ± 0.2; 156 Gp2: 1184.2 ± 30.2; 29.2 ± 0.3; 46	Gp 1: ≥50% HM (MOM) [89% at 4 wk] Gp 2: <50% HM (MOM) [10.7% at 4 wk]	C3: Gp1 vs. Gp2	SS: <72 h of birth SE: 14 d from study start	NEC (clinical ± radiographic evidence) Sepsis ((+)ve blood culture) BPD (not defined) ROP (all stages); Severe ROP (requiring laser surgery)	Moderate (some differences between gps)
Sisk (2017) [64] USA	Cohort	563 infants Whole cohort: 1021 ± 285; 27.9 ± 2.4 *n* = Gp1, 299; Gp2, 139; Gp3, 113	Gp1: ≥50% MOM [97 (82, 100) MOM] Gp2: ≥50% PDHM [86% (74, 96) DHM] Gp3: ≥50% PTF [9% (0, 26) MOM]	C3: Gps1,2 vs. Gp3 C4: Gp1 vs. Gp2	SS: Birth SE 34 wk PMA	NEC (≥Bell’s stage 2) Severe NEC (requiring surgery) Sepsis ((+)ve blood culture) BPD (O_2_ @ 36 wk) Severe ROP (Grade 3 or 4)	Low
Spiegler (2016) [66] Germany	Cohort	1433 infants Gp1: 1080 (830, 1330); 28.7 (26.6, 30.1); 239 Gp2: 1100 (865, 1340); 29.0 (26.9,3 0.0); 223 Gp3: 1050 (805, 1295); 28.4 (26.6, 30.0); 971	Gp1: EPTF [0%] Gp2: EHM (MOM) [100%] Gp3: Mixed feeding [NR]	C1 Gp2 vs. Gp1: C2: Gps2,3 vs. Gp1 C3: Gp2 vs. Gp3	Duration of neonatal admission	NEC (Bell’s stage 2/3) Severe NEC (requiring surgery) BPD (O_2_ @ 36 wk) ROP (all stages); Severe ROP (Stage3/4)	Low
Stock (2015) [20] Austria	Interrupted time series	323 infants Gp1:1226.8 ± 382; 29.5 (27.7, 30.7); 159 Gp2: 1271.3 ± 412; 30 (28.2, 31.2); 164	Gp 1: Pasteurisation era (2008–2010) [NR] Gp 2: Unpasteurised era (2010–2013) NR]	C4: Gp2 vs. Gp1	Duration of neonatal admission.	NEC (Bell’s criteria) Sepsis (clinical signs	Low
Tanaka (2009) [22] Japan	Cohort	18 infants Gp1: 1016.4 ± 302.2; 28.7 ± 3.2; 10 Gp2: 1188.0 ± 296.3; 30.7 ± 1.6; 8	Gp 1: >80% HM in first month Gp 2: <80% HM in first month	C3: Gp 1 vs. Gp2	Group allocation based on feeds within first month of life with outcome follow-up at 5 years	NEC (not defined) BPD (not defined) ROP (not defined) Neurodevelopment (Kaufman Assessment Battery for Children, + others)	High (unclear recruitment, some loss to FU)
Verd (2015) [23] Spain	Cohort	201 infants Gp1: 800 (410, 995); 26.4 (23, 33.7); 148 Gp2: 830 (440, 998); 27.1 (23.7, 34.1); 53	Gp 1: EHM (MOM + DM) [100%] Gp 2: Mixed (MOM + PTF) [NR]	C3: Gp 1 vs. Gp 2	Duration of neonatal admission	NEC (not defined) Severe NEC (requiring surgery) Sepsis ((+)ve blood culture) ROP (any stage) Severe ROP (requiring surgery)	Low
Were (2006) [67] Africa	Cohort	120 infants Whole cohort: 1420 ± 93; 32.5 ± 2.4 *n* = Gp1, 54, Gp2, 27, Gp3, 39	Gp1: EHM [100%] Gp2: EPTF [0%] Gp3: Mixed feeds [NR]	C3: Synthesised narratively	Duration of neonatal admission	Neurodevelopment (Dorothy Egan’s Model, Saigal and Rosenbaum’s method)	High (unclear recruitment, some loss to FU)

Data presented as mean ± SD or median (IQR) unless otherwise stated. * As reported in original article (assume misprint). Abbreviations: Ax, assessment; BovF, bovine fortifier; BPD, bronchopulmonary dysplasia; BSID, Bayley Scales of Infant Development; BW, birth weight; C1–4, comparison 1–4; CA, corrected age; CSF, cerebrospinal fluid; d, days; DC, discharge; EHM, exclusive human milk; EPTF, exclusive preterm formula; FU, follow-up; GA, gestational age; Gp, group; h, hour; HM, human milk; HMDF, human milk derived fortifier; HR, hazard ratio; LOS, late onset sepsis; m, month; MOM, mother’s own milk; NEC, necrotising enterocolitis; NICU, neonatal intensive care unit; O_2_, oxygen; NR, not reported; PDM, pasteurised donor milk; PMA, postmenstrual age; PTF, preterm formula; ROP, retinopathy of prematurity; RT, randomised trial; SE, study end; SS, study start; VLBW, very low birth weight; wk, weeks.

**Table 2:** Summary of Findings.

	Comparison	EHM vs. EPTF RR or MD (95% CI); N Participants (Studies), *I*^2^ GRADE Certainty of Evidence Interpretation and Absolute effect (95% CI)	Any HM vs. EPTF RR or MD (95% CI); N Participants (Studies), *I*^2^ GRADE Certainty of Evidence Interpretation and Absolute Effect (95% CI)	High vs. Low Dose HM RR or MD (95% CI); N Participants (Studies), *I*^2^ GRADE Certainty of Evidence Interpretation and Absolute Effect (95% CI)	Unpasteurised vs. Pasteurised RR or MD (95% CI); N Participants (Studies), *I*^2^ GRADE Certainty of Evidence Interpretation and Absolute Effect (95% CI)
Outcome					
NEC		RTs RR 0.17 (0.02, 1.32); 53, (1 RT) Certainty: Low Obs RR 0.22 (0.09, 0.54), 933, (3 studies), *I*^2^ = 0% Certainty: Moderate Interpretation Possible reduction in any NEC Absolute risk reduction of 4.3% (from 2.5 to 5 fewer/100)	Obs RR 0.51 (0.35, 0.76); 3783, (9 studies), *I*^2^ = 7% Certainty: Moderate Interpretation Clear reduction in any NEC Absolute reduction of 3.6% (from 1.8 to 4.8 fewer/100)	RTs RR 0.59 (0.39, 0.89) fixed effects; 1116, (4 RTs), *I*^2^ = 50% Certainty: Moderate Obs RR: 0.53 (0.42, 0.67); 8778 (22 studies), *I*^2^ = 28% Certainty: Moderate Interpretation Clear reduction in any NEC Absolute risk reduction between 3.8 and 4.3 % (from 0.2 more to 6.8 fewer/100)	RT RR 1.45 (0.64, 3.30); 303 (1 RT) Certainty: Low Obs RR 1.28 (0.68, 2.43), 1894 (6 studies), *I*^2^ = 30% Certainty: Very low Interpretation Inconclusive
NEC requiring surgery		RT RR 0.09 (0.01, 1.64); 53, (1 RT) Certainty: Low Obs RR 0.22 (0.03, 1.86), 444, (1 study) Certainty: Very low Interpretation Inconclusive	Obs RR 0.30 (0.05, 1.76); 1420, (3 studies), *I*^2^ = 50% Certainty: Very low Interpretation Inconclusive	RTs RR 0.36 (0.06, 2.04) 580, (2 RTs), *I*^2^ = 66% Certainty: Low Obs RR: 0.51 (0.33, 0.79); 2964 (6 studies), *I*^2^ = 0% Certainty: Moderate Interpretation Possible reduction in severe NEC Absolute reduction (obs studies) 1.8% (from 0.8 to 2.4 fewer/100)	RT RR 0.11 (0.01, 2.06); 303 (1 RT) Certainty: Low Obs RR 1.59 (0.14, 17.85), 530 (2 studies), *I*^2^ = 42% Certainty: Very low Interpretation Inconclusive
LOS		RTs RR 0.7 (0.47, 1.03); 53 (1 RT) Certainty: Low Obs RR 0.71 (0.49, 1.05); 776 (3 studies), *I*^2^= 0% Certainty: Low Interpretation Possible reduction in LOS ^1^ Absolute reduction from RT of 23.8% (from 42 fewer to 2.4 more cases/100) and from observational studies 5% (from 0.9 more to 8.9 fewer cases/100)	Obs RR 0.95 (0.67, 1.34); 2497 (8 studies), *I*^2^ = 59% Certainty: Very low Interpretation Inconclusive	RTs RR 1.07 (0.89, 1.28); 1186 (4 RTs), *I*^2^ = 0% Certainty: Moderate Obs RR 0.71 (0.56, 0.9); 6521 (18 studies), *I*^2^ = 74% Certainty: Very low Interpretation Inconclusive	RT RR 0.71 (0.43, 1.18); 303 (1 RT) Certainty: Moderate Obs RR 1.05 (0.86, 1.27); 1875 (5 studies), *I*^2^ = 14% Certainty: Very low Interpretation Possibly no effect
BPD		Obs RR 0.94 (0.26, 3.41); 706 (2 studies), *I*^2^ = 79% Certainty: Very low Interpretation Inconclusive	Obs RR 1.02 (0.83, 1.27); 3703 (6 studies), *I*^2^ = 54% Certainty: Very low Interpretation Inconclusive	RTs RR 0.95 (0.73, 1.25); 1075 (4 RTs), *I*^2^ = 42% Certainty: Low Obs RR 0.84 (0.73, 0.96); 7023 (18 studies), *I*^2^ = 53% Certainty: Very low Interpretation Inconclusive	RTs RR 0.69 (0.43, 1.1); 303 (1 RT) Certainty: Low Obs RR 1.01 (0.72, 1.43) 1644 (5 studies), *I*^2^ = 39% Certainty: Very low Interpretation Inconclusive
ROP		RT RR 1.32 (0.5, 3.52); 53 (1 RT) Certainty: Low Obs RR 0.65 (0.31, 1.34); 1256 (4 studies), *I*^2^ = 84% Certainty: Very low Interpretation Insufficient evidence to draw conclusion	Obs RR 1.08 (0.79, 1.48); 3576 (6 studies), *I*^2^ = 75% Certainty: Very low Interpretation Inconclusive	RTs RR 1.14 (0.86, 1.5); 1071 (4 RTs), I^2^=0% Certainty: Moderate Obs RR 0.82 (0.70, 0.96); 6302 (18 studies), I^2^ = 43% Certainty: Very low Interpretation Inconclusive	RT RR 0.89 (0.35, 2.26); 303 (1RT) Certainty: Low Obs RR 0.89 (0.33, 2.38); 681 (3 studies), *I*^2^ = 73% Certainty: Very low Interpretation Inconclusive
Severe ROP^1^		Obs RR 0.23 (0.07, 0.73); 1012 (3 studies), *I*^2^ = 57% Certainty: Low Interpretation Possible reduction Absolute reduction of 7.6% (from 2.7to 9.1 fewer/100)	Obs RR 0.81 (0.42, 1.56); 2553 (3 studies), *I*^2^ = 74% Certainty: Very low Interpretation Inconclusive	RTs RR 1.15 (0.66, 2.02); 536 (2 RTs), *I*^2^ = 0% Certainty: Low Obs RR 0.63 (0.46, 0.87); 5224 (13 studies), *I*^2^ = 22% Certainty: Low Interpretation Inconclusive	RT RR 0.89 (0.35, 2.26); 303 (1RT) Certainty: Low Obs RR 0.81 (0.13, 5.08); 589 (2 studies), *I*^2^ = 86% Certainty: Very low Interpretation Inconclusive
Neurodevelopment		No studies identified	0 to <18 months Cognition Obs MD 9 higher (1.42 fewer to 16.58 higher); 39 (1 study) Certainty: Very low 18 to <36 months Cognition Obs MD 2.01 higher (1.35 lower to 5.36 higher); 1744 (3 studies) *I*^2^ = 49% Certainty: Very low Motor Obs MD 0.8 lower (6.02 lower, 4.42 higher); 1744 (3 studies) *I*^2^ = 77% Certainty: Very low Interpretation Inconclusive	0 to <18 months Cognition Obs MD 0.67 higher (2.68 lower to 4.03 higher); 684 (5 studies) *I*^2^ = 58% Certainty: Very low Motor Obs MD 0.33 lower (4.8 lower to 4.14 higher) 684 (5 studies) *I*^2^ = 69% Certainty: Very low 18 to <36 months Cognition RT MD 1.6 lower (5.95 lower to 2.75 higher); 299 (1 RT) Certainty: Moderate Cognition Obs MD 0.59 lower (3.41 lower to 2.24 higher); 722 (4 studies) *I*^2^ = 9% Certainty: Very low Motor RT MD 2.2 lower (6.42 lower to 2.02 higher); 299 (1 RT) Certainty: Moderate Motor Obs MD 1.94 lower (4.78 lower to 0.9 higher); 722 (4 studies) *I*^2^ = 9% Certainty: Very low >3 years Cognition Obs MD 6.4 higher (5.8 lower to 18.6 higher); 18 (1 study) Certainty: Very low Interpretation Inconclusive	No studies identified

Footnotes: ^1^ Although the RT and meta-analysis of observational studies did not reach significance, the CIs neared 1 and, as such, we conclude there is a possible reduction in the incidence of LOS. Abbreviations: BPD. Bronchopulmonary dysplasia; EHM, exclusive human milk; EPTF, exclusive preterm formula; HM, human milk, LOS, late onset sepsis; MD, mean difference; NEC, necrotising enterocolitis; Obs, observational studies; RR, relative risk; ROP, retinopathy of prematurity; RTs, randomised trials.

## Supplementary Materials

The following are available online at http://www.mdpi.com/2072-6643/10/6/707/s1. Record of database searches, Figure S1: Selection of studies, Figure S2: Forest plot of relative risk for the association of human milk and severe necrotising enterocolitis, Figure S3: Forest plot of relative risk for the association between human milk and severe retinopathy of prematurity, Table S1: Ovid Medline search strategy, Table S2: CINAHL search strategy, Table S3: any NEC, Summary of findings, Table S4: severe NEC, Summary of findings, Table S5: LOS, Summary of findings, Table S6: BPD, Summary of findings, Table S7, ROP Summary of findings, Table S8: severe ROP Summary of findings, Table S9: Neurodevelopment Summary of findings.
