# Supplementary material for: A Systematic Review and Meta-Analysis of Human Milk Feeding and Morbidity in Very Low Birth Weight Infants
Source: Nutrients. 2018 May 31;10(6):707. doi: 10.3390/nu10060707 (PMC6024377; doi:10.3390/nu10060707)
Supplement: Supplementary file 1 [file nutrients-10-00707-s001.pdf]

Supplementary Materials

Table of Contents

Record of database search strategies 2
Ovid MEDLINE 2
PubMed 3
CINAHL 4
Scopus 7
Cochrane Central Register of Controlled Trials 8
References - excluded studies 10

Figures

Figure S1. PRISMA diagram - selection of studies 9
Figure S2. Forest plot of relative risk for the association of human milk and severe necrotising enterocolitis 15
Figure S3. Forest plot of relative risk for the association between human milk and severe retinopathy of prematurity 21

Tables

Table S1. Ovid MEDLINE search strategy 2
Table S2. CINAHL search strategy 4
Table S3. Any necrotising enterocolitis: summary of findings 16
Table S4. Severe necrotising enterocolitis: summary of findings 17
Table S5. Late onset sepsis: summary of findings 18
Table S6. Bronchopulmonary dysplasia: summary of findings 19
Table S7. Retinopathy of prematurity: summary of findings 20
Table S8. Severe retinopathy of prematurity: summary of findings 22
Table S9. Neurodevelopment: summary of findings 23

Record of database search strategies - run 13/6/17

Ovid MEDLINE(R) Epub Ahead of Print, In-Process & Other Non-Indexed Citations, Ovid MEDLINE(R) Daily, Ovid MEDLINE and Versions(R)

Table S1. Ovid MEDLINE search strategy

#	Searches	Results
1	Infant, Premature/ OR Infants, Extremely premature/ OR Infant, Low birth weight/ OR Infant, Very Low Birth Weight/ OR Intensive care units, Neonatal/ OR Intensive care, Neonatal/ OR Premature birth/	83735
2	(((low birthweight OR low birth weight OR VLBW OR ELBW OR Prematur* OR Preterm OR Pre-term) adj2 (Infant* OR Neonat* OR newborn* OR new-born* OR baby* OR babies)) OR neonatal intensive care OR NICU).tw,kw.	73321
3	or/1-2	111350
4	Milk, Human/ OR Breast feeding/ OR Milk banks/ OR Breast milk expression/	46962
5	((breastmilk OR (human OR breast OR mother* OR maternal OR express* OR donor* OR donated OR bank*)) adj3 milk*).tw,kw.	23289
6	(breastfeed* OR breastfed OR ((breast OR HM) adj (fed OR feed*))).tw,kw.	36982
7	((EHM OR MOM OR PDM) and milk).tw,kw.	45
8	Enteral Nutrition/	18724
9	(Enteral* adj1 (nutrition* OR support OR feed*)).tw,kw.	12409
10	or/4-9	90007
11	infant, premature, diseases/	19459
12	((prematur* OR preterm OR pre-term) adj2 disease*).tw,kw.	3129
13	Enterocolitis, Necrotizing/	2706
14	(Necroti?ing enterocolitis OR NEC).tw,kw.	8044
15	Bronchopulmonary dysplasia/ OR Respiratory Distress Syndrome, Newborn/	15536
16	(Bronchopulmonary dysplasia OR broncho-pulmonary dysplasia OR BPD OR lung disease* OR lung disorder* OR immature lung* OR respiratory distress).tw,kw.	92321
17	"retinopathy of prematurity"/	5206
18	("Retinopathy of prematurity" OR ROP).tw,kw.	6468
19	exp Sepsis/ OR exp Infection/	726204
20	(sepsis OR sepses OR septic OR septic?emi* OR infect* OR bacteria* OR bacter?emi* OR microorganism* OR micro-organism* OR blood poisoning* OR pyoh?emi* OR py?emi*).tw,kw.	2051314
21	Child development/ OR Brain/ OR Intelligence/ OR Cognition/ OR Executive function/ OR Motor skills/ OR Motor activity/	669535
22	Intelligence tests/ OR Neuropsychological tests/	95266
23	((infan* OR child*) adj2 development*).tw,kw.	26347
24	((brain OR neurologic* OR cognitive* OR intellectual* OR motor OR psychomotor) adj2 development*).tw,kw.	37412
25	(neurodevelopment* OR neuro-development* OR neurocognitive* OR neuro-cognitive OR neurobehavio?r* OR neuro-behavio?r* OR neuropsycholog* OR neuro-psycholog* OR cognition OR intelligence OR executive function).tw,kw.	166261
26	or/11-25	3318491
27	3 and 10 and 26	2994
28	exp animals/ not (exp animals/ and humans/)	4417385

29	27 not 28	2945
30	limit 29 to english language	2677
31	limit 30 to yr="1990 -Current"	2441

PubMed (non-indexed subset only)

N=373

((((“low birthweight”[tiab] OR “low birth weight”[tiab] OR VLBW[tiab] OR ELBW[tiab] OR Prematur*[tiab] OR Preterm[tiab] OR “Pre-term”[tiab]) AND (Infant*[tiab] OR Neonat*[tiab] OR newborn*[tiab] OR “new-born*”[tiab] OR baby*[tiab] OR babies[tiab])) OR “neonatal intensive care”[tiab] OR NICU[tiab]) AND (((breastmilk[tiab] OR (human[tiab] OR breast[tiab] OR mother*[tiab] OR maternal[tiab] OR express*[tiab] OR donor*[tiab] OR donated[tiab] OR bank*[tiab])) AND milk*[tiab]) OR (breastfeed*[tiab] OR breastfed[tiab] OR ((breast[tiab] OR HM[tiab]) AND (fed[tiab] OR feed*[tiab]))) OR ((EHM[tiab] OR MOM[tiab] OR PDM[tiab]) AND milk[tiab]) OR (Enteral*[tiab] AND (nutrition*[tiab] OR support[tiab] OR feed*[tiab]))) AND (((prematur*[tiab] OR preterm[tiab] OR “pre-term” [tiab]) AND disease*[tiab]) OR “Necrotising enterocolitis”[tiab] OR “Necrotizing enterocolitis”[tiab] OR NEC[tiab] OR “Bronchopulmonary dysplasia”[tiab] OR “broncho-pulmonary dysplasia”[tiab] OR BPD[tiab] OR “lung disease*”[tiab] OR “lung disorder*”[tiab] OR “immature lung*”[tiab] OR “respiratory distress”[tiab] OR "Retinopathy of prematurity"[tiab] OR ROP[tiab] OR sepsis[tiab] OR sepses[tiab] OR septic[tiab] OR septicemi* [tiab] OR septicaemi*[tiab] OR infect*[tiab] OR bacteria*[tiab] OR bacteremi*[tiab] OR bacteraemi*[tiab] OR microorganism*[tiab] OR “micro-organism*”[tiab] OR “blood poisoning*”[tiab] OR pyohemi*[tiab] OR pyohaemi*[tiab] OR pyemi*[tiab] OR pyaemi*[tiab] OR ((brain[tiab] OR neurologic*[tiab] OR cognitive*[tiab] OR intellectual*[tiab] OR motor[tiab] OR psychomotor[tiab]) AND development*[tiab]) OR ((infan*[tiab] OR child*[tiab]) AND development*[tiab]) OR neurodevelopment*[tiab] OR “neuro-development*”[tiab] OR neurocognitive*[tiab] OR “neuro-cognitive”[tiab] OR neurobehavior*[tiab] OR neurobehaviour*[tiab] OR “neuro-behavior*”[tiab] OR “neuro-behaviour*”[tiab] OR neuropsycholog*[tiab] OR “neuro-psycholog*”[tiab] OR cognition[tiab] OR intelligence[tiab] OR “executive function”[tiab]) AND English[la] AND 1990:2017[dp]) NOT Medline[sb]

CINAHL (EBSCOhost)

Table S2. CINAHL search strategy

#	Query	Limiters/Expanders	Results
S1	(MH "Infant, Premature") OR (MH "Infant, Low Birth Weight") OR (MH "Infant, Very Low Birth Weight")	Search modes - Boolean/Phrase	17,057
S2	(MH "Childbirth, Premature")	Search modes - Boolean/Phrase	5,058
S3	(MH "Intensive Care, Neonatal") OR (MH "Intensive Care Units, Neonatal")	Search modes - Boolean/Phrase	10,284
S4	TI ((((“low birthweight” OR “low birth weight” OR VLBW OR ELBW OR Prematur* OR Preterm OR “Pre-term”) N1 (Infant* OR Neonat* OR newborn* OR “new-born*” OR baby* OR babies)) OR “neonatal intensive care” OR NICU)) OR AB ((((“low birthweight” OR “low birth weight” OR VLBW OR ELBW OR Prematur* OR Preterm OR “Pre-term”) N1 (Infant* OR Neonat* OR newborn* OR “new-born*” OR baby* OR babies)) OR “neonatal intensive care” OR NICU))	Search modes - Boolean/Phrase	17,903
S5	S1 OR S2 OR S4	Search modes - Boolean/Phrase	28,457
S6	(MH "Milk, Human") OR (MH "Donor Milk")	Search modes - Boolean/Phrase	3,094
S7	(MH "Milk Banks")	Search modes - Boolean/Phrase	289
S8	(MH "Breast Pumps")	Search modes - Boolean/Phrase	289
S9	(MH "Breast Feeding") OR (MH "Milk Expression")	Search modes - Boolean/Phrase	13,429
S10	TI ((((breastmilk OR (human OR breast OR mother* OR maternal OR express* OR donor* OR donated OR bank*)) N2 milk*) OR (breastfeed* OR breastfed OR ((breast OR HM) N0 (fed OR feed*))) OR ((EHM OR MOM OR PDM) AND milk) OR (Enteral* N0 (nutrition* OR support OR feed*)))) OR AB ((((breastmilk OR (human OR breast OR mother* OR maternal OR express* OR donor* OR donated OR bank*)) N2 milk*) OR (breastfeed* OR breastfed OR ((breast OR HM) N0 (fed OR feed*))) OR ((EHM OR MOM OR PDM) AND milk) OR (Enteral* N0 (nutrition* OR support OR feed*))))	Search modes - Boolean/Phrase	16,416
S11	(MH "Enteral Nutrition")	Search modes - Boolean/Phrase	5,571
S12	S6 OR S7 OR S8 OR S9 OR S10 OR S11	Search modes - Boolean/Phrase	25,094
S13	(MH "Infant, Newborn, Diseases") OR (MH "Bronchopulmonary Dysplasia") OR (MH "Enterocolitis, Necrotizing") OR (MH "Respiratory Distress Syndrome") OR (MH "Retinopathy of Prematurity") OR (MH "Neonatal Sepsis")	Search modes - Boolean/Phrase	6,483

#	Query	Limiters/Expanders	Results
S14	(MH "Infection+")	Search modes - Boolean/Phrase	90,193
S15	(MH "Child Development") OR (MH "Infant Development")	Search modes - Boolean/Phrase	14,958
S16	(MH "Brain")	Search modes - Boolean/Phrase	21,857
S17	(MH "Intelligence")	Search modes - Boolean/Phrase	2,999
S18	(MH "Cognition")	Search modes - Boolean/Phrase	23,286
S19	(MH "Executive Function")	Search modes - Boolean/Phrase	604
S20	(MH "Motor Skills") OR (MH "Psychomotor Performance")	Search modes - Boolean/Phrase	10,397
S21	(MH "Motor Activity")	Search modes - Boolean/Phrase	4,705
S22	(MH "Intelligence Tests")	Search modes - Boolean/Phrase	4,483
S23	(MH "Neuropsychological Tests")	Search modes - Boolean/Phrase	19,216
S24	TI ((((prematur* OR preterm OR “pre-term”) N1 disease*) OR “Necrotising enterocolitis” OR “Necrotizing enterocolitis” OR NEC OR “Bronchopulmonary dysplasia” OR “broncho-pulmonary dysplasia” OR BPD OR “lung disease*” OR “lung disorder*” OR “immature lung*” OR “respiratory distress” OR "Retinopathy of prematurity" OR ROP OR sepsis OR sepses OR septic OR septicemi* OR septicaemi* OR infect* OR bacteria* OR bacteremi* OR bacteraemi* OR microorganism* OR “micro-organism*” OR “blood poisoning*” OR pyohemi* OR pyohaemi* OR pyemi* OR pyaemi* OR ((brain OR neurologic* OR cognitive* OR intellectual* OR motor OR psychomotor) N1 development*) OR ((infan* OR child*) N1 development*) OR neurodevelopment* OR “neuro-development*” OR neurocognitive* OR “neuro-cognitive” OR neurobehavior* OR neurobehaviour* OR “neuro-behavior*” OR “neuro-behaviour*” OR neuropsycholog* OR “neuro-psycholog*” OR cognition OR intelligence OR “executive function”)) OR AB ((((prematur* OR preterm OR “pre-term”) N1 disease*) OR “Necrotising enterocolitis” OR “Necrotizing enterocolitis” OR NEC OR “Bronchopulmonary dysplasia” OR “broncho-pulmonary dysplasia” OR BPD OR “lung disease*” OR “lung disorder*” OR “immature lung*” OR “respiratory distress” OR "Retinopathy of prematurity" OR ROP OR sepsis OR sepses OR septic OR septicemi* OR septicaemi* OR infect* OR bacteria* OR bacteremi* OR bacteraemi* OR microorganism* OR “micro-organism*” OR “blood poisoning*” OR pyohemi* OR pyohaemi* OR pyemi* OR pyaemi* OR ((brain OR	Search modes - Boolean/Phrase	177,981

#	Query	Limiters/Expanders	Results
	neurologic* OR cognitive* OR intellectual* OR motor OR psychomotor) N1 development*) OR ((infan* OR child*) N1 development*) OR neurodevelopment* OR "neuro-development*" OR neurocognitive* OR "neuro-cognitive" OR neurobehavior* OR neurobehaviour* OR "neuro-behavior*" OR "neuro-behaviour*" OR neuropsycholog* OR "neuro-psycholog*" OR cognition OR intelligence OR "executive function"))		
S25	S13 OR S14 OR S15 OR S16 OR S17 OR S18 OR S19 OR S20 OR S21 OR S22 OR S23 OR S24	Search modes - Boolean/Phrase	291,095
S26	S5 AND S12 AND S25	Search modes - Boolean/Phrase	880
S27	S5 AND S12 AND S25	Narrow by Language: - english<br>Search modes - Boolean/Phrase	858
S28	S5 AND S12 AND S25	Limiters - Published Date: 19900101-20171231<br>Narrow by Language: - english<br>Search modes - Boolean/Phrase	694

Scopus

N=2773

TITLE-ABS-KEY(((("low birthweight" OR "low birth weight" OR VLBW OR ELBW OR Prematur* OR Preterm OR "Pre-term") W/1 (Infant* OR Neonat* OR newborn* OR "new-born*" OR baby* OR babies)) OR "neonatal intensive care" OR NICU) AND (((breastmilk OR (human OR breast OR mother* OR maternal OR express* OR donor* OR donated OR bank*)) W/2 milk*) OR (breastfeed* OR breastfed OR ((breast OR HM) W/0 (fed OR feed*))) OR ((EHM OR MOM OR PDM) AND milk) OR (Enteral* W/0 (nutrition* OR support OR feed*))) AND (((prematur* OR preterm OR "pre-term") W/1 disease*) OR "Necrotising enterocolitis" OR "Necrotizing enterocolitis" OR NEC OR "Bronchopulmonary dysplasia" OR "broncho-pulmonary dysplasia" OR BPD OR "lung disease*" OR "lung disorder*" OR "immature lung*" OR "respiratory distress" OR "Retinopathy of prematurity" OR ROP OR sepsis OR sepses OR septic OR septicemi* OR septicaemi* OR infect* OR bacteria* OR bacteremi* OR bacteraemi* OR microorganism* OR "micro-organism*" OR "blood poisoning*" OR pyohemi* OR pyohaemi* OR pyemi* OR pyaemi* OR ((brain OR neurologic* OR cognitive* OR intellectual* OR motor OR psychomotor) W/1 development*) OR ((infan* OR child*) W/1 development*) OR neurodevelopment* OR "neuro-development*" OR neurocognitive* OR "neuro-cognitive" OR neurobehavior* OR neurobehaviour* OR "neuro-behavior*" OR "neuro-behaviour*" OR neuropsycholog* OR "neuro-psycholog*" OR cognition OR intelligence OR "executive function")) AND (LIMIT-TO (DOCTYPE,"ar") OR LIMIT-TO (DOCTYPE,"re") OR LIMIT-TO (DOCTYPE,"ip")) AND (LIMIT-TO (LANGUAGE,"English")) AND (EXCLUDE (PUBYEAR,1989) OR EXCLUDE (PUBYEAR,1988) OR EXCLUDE (PUBYEAR,1987) OR EXCLUDE (PUBYEAR,1986) OR EXCLUDE (PUBYEAR,1985) OR EXCLUDE (PUBYEAR,1984) OR EXCLUDE (PUBYEAR,1983) OR EXCLUDE (PUBYEAR,1982) OR EXCLUDE (PUBYEAR,1981) OR EXCLUDE (PUBYEAR,1980) OR EXCLUDE (PUBYEAR,1979) OR EXCLUDE (PUBYEAR,1978) OR EXCLUDE (PUBYEAR,1977) OR EXCLUDE (PUBYEAR,1976) OR EXCLUDE (PUBYEAR,1975) OR EXCLUDE (PUBYEAR,1974) OR EXCLUDE (PUBYEAR,1973) OR EXCLUDE (PUBYEAR,1972) OR EXCLUDE (PUBYEAR,1971) OR EXCLUDE (PUBYEAR,1970) OR EXCLUDE (PUBYEAR,1969) OR EXCLUDE (PUBYEAR,1968) OR EXCLUDE (PUBYEAR,1967) OR EXCLUDE (PUBYEAR,1966) OR EXCLUDE (PUBYEAR,1965) OR EXCLUDE (PUBYEAR,1964) OR EXCLUDE (PUBYEAR,1963) OR EXCLUDE (PUBYEAR,1959) OR EXCLUDE (PUBYEAR,1957) OR EXCLUDE (PUBYEAR,1954) OR EXCLUDE (PUBYEAR,1948) OR EXCLUDE (PUBYEAR,1946) OR EXCLUDE (PUBYEAR,1939))

Cochrane Central Register of Controlled Trials (Issue 5 of 12, May 2017)

N=311

(((“low birthweight” OR “low birth weight” OR VLBW OR ELBW OR Prematur* OR Preterm OR “Pre-term”) NEAR/1 (Infant* OR Neonat* OR newborn* OR “new-born*” OR baby* OR babies)) OR “neonatal intensive care” OR NICU) AND (((breastmilk OR (human OR breast OR mother* OR maternal OR express* OR donor* OR donated OR bank*)) NEAR/2 milk*) OR (breastfeed* OR breastfed OR ((breast OR HM) NEAR/0 (fed OR feed*))) OR ((EHM OR MOM OR PDM) AND milk) OR (Enteral* NEAR/0 (nutrition* OR support OR feed*))) AND (((prematur* OR preterm OR “pre-term”) NEAR/1 disease*) OR “Necrotising enterocolitis” OR “Necrotizing enterocolitis” OR NEC OR “Bronchopulmonary dysplasia” OR “broncho-pulmonary dysplasia” OR BPD OR “lung disease*” OR “lung disorder*” OR “immature lung*” OR “respiratory distress” OR "Retinopathy of prematurity" OR ROP OR sepsis OR sepses OR septic OR septicemi* OR septicaemi* OR infect* OR bacteria* OR bacteremi* OR bacteraemi* OR microorganism* OR “micro-organism*” OR “blood poisoning*” OR pyohemi* OR pyohaemi* OR pyemi* OR pyaemi* OR ((brain OR neurologic* OR cognitive* OR intellectual* OR motor OR psychomotor) NEAR/1 development*) OR ((infan* OR child*) NEAR/1 development*) OR neurodevelopment* OR “neuro-development*” OR neurocognitive* OR “neuro-cognitive” OR neurobehavior* OR neurobehaviour* OR “neuro-behavior*” OR “neuro-behaviour*” OR neuropsycholog* OR “neuro-psycholog*” OR cognition OR intelligence OR “executive function”)

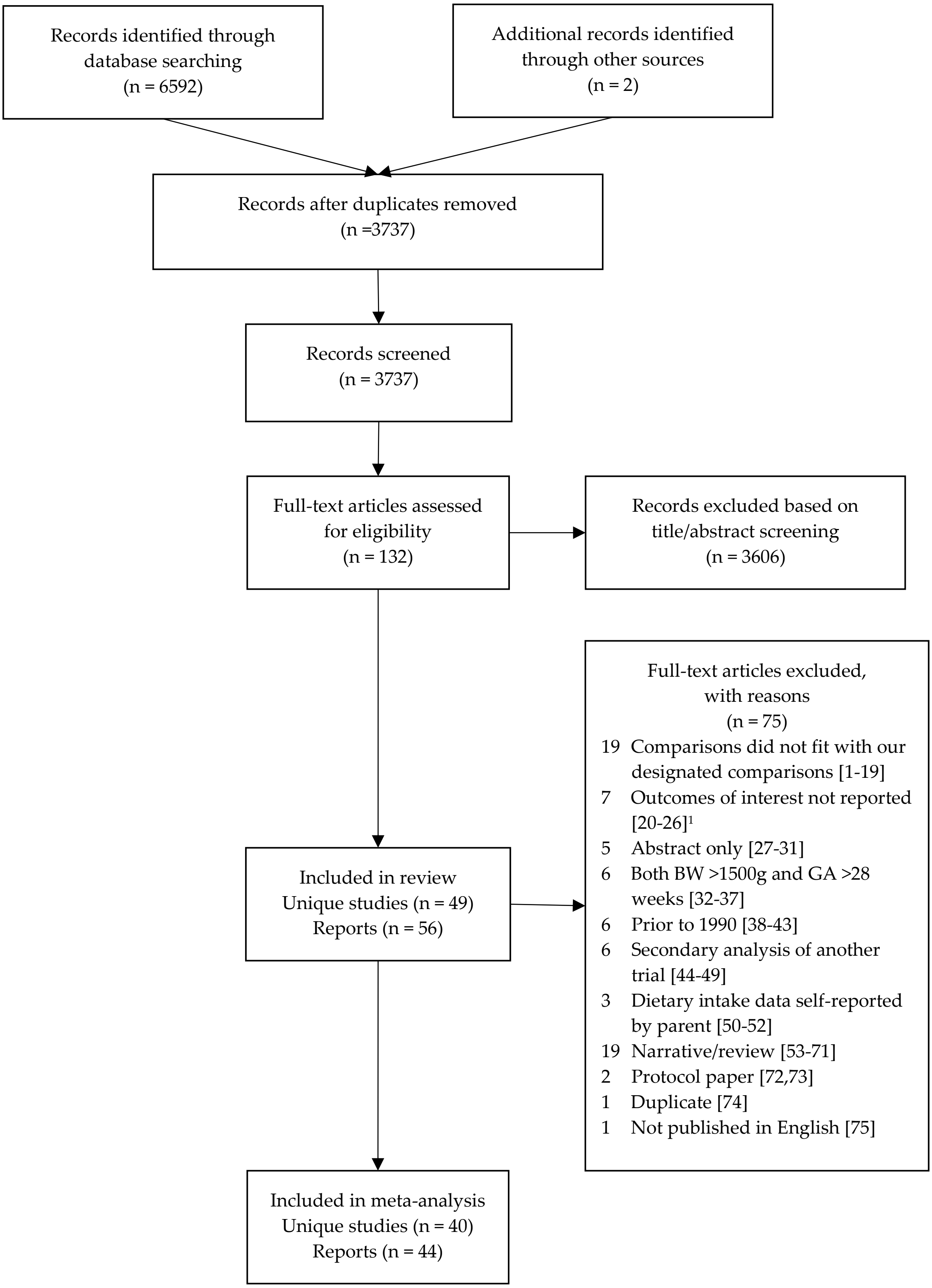

Figure S1. PRISMA diagram - selection of studies

[1] This includes one study which reported combined outcomes of interest [21]

References - excluded studies

1. In the News - News from the National Institute of Nursing Research. Breast milk better for preemies. *Am J Nurs.* **2008**, *108*, 22.
2. Kanmaz, H.G.; Mutlu, B.; Canpolat, F.E.; Erdeve, O.; Oguz, S.S.; Uras, N.; Dilmen, U. Human milk fortification with differing amounts of fortifier and its association with growth and metabolic responses in preterm infants. *J Hum Lact* **2013**, *29*, 400-405.
3. Boo, N.Y.; Puah, C.H.; Lye, M.S. The role of expressed breastmilk and continuous positive airway pressure as predictors of survival in extremely low birthweight infants. *J. Trop. Pediatr.* **2000**, *46*, 15-20.
4. Butler, T.J.; Szekely, L.J.; Grow, J.L. A standardized nutrition approach for very low birth weight neonates improves outcomes, reduces cost and is not associated with increased rates of necrotizing enterocolitis, sepsis or mortality. *J. Perinatol.* **2013**, *33*, 851-857.
5. Chye, J.K.; Lim, C.T.; Leong, H.L.; Wong, P.K. Retinopathy of prematurity in very low birth weight infants. *Ann Acad Med Singapore* **1999**, *28*, 193-198.
6. Collins, C.T.; Gibson, R.A.; Anderson, P.J.; McPhee, A.J.; Sullivan, T.R.; Gould, J.F.; Ryan, P.; Doyle, L.W.; Davis, P.G.; McMichael, J.E., *et al.* Neurodevelopmental outcomes at 7 years' corrected age in preterm infants who were fed high-dose docosahexaenoic acid to term equivalent: A follow-up of a randomised controlled trial. *BMJ Open.* **2015**, *5*, e007314.
7. Filipouski, G.; Silveira, R.; Procianoy, R. Influence of perinatal nutrition and gestational age on neurodevelopment of very low-birth-weight preterm infants. *Am. J. Perinatol.* **2013**, *30*, 673-680.
8. Kantorowska, A.; Wei, J.C.; Cohen, R.S.; Lawrence, R.A.; Gould, J.B.; Lee, H.C. Impact of donor milk availability on breast milk use and necrotizing enterocolitis rates. *Pediatrics.* **2016**, *137*, e20153123.
9. Khorana, M.; Jiamsajjamongkhon, C. Pilot study on growth parameters and nutritional biochemical markers in very low birth weight preterm infants fed human milk fortified with either human milk fortifier or post discharge formula. *J. Med. Assoc. Thailand* **2014**, *97*, S164-S175.
10. Olsen, I.E.; Richardson, D.K.; Schmid, C.H.; Ausman, L.M.; Dwyer, J.T. Intersite differences in weight growth velocity of extremely premature infants. *Pediatrics.* **2002**, *110*, 1125-1132.
11. Profit, J.; Zupancic, J.A.; Gould, J.B.; Pietz, K.; Kowalkowski, M.A.; Draper, D.; Hysong, S.J.; Petersen, L.A. Correlation of neonatal intensive care unit performance across multiple measures of quality of care. *JAMA Pediatr.* **2013**, *167*, 47-54.
12. Vohr, B.R.; Wright, L.L.; Poole, W.K.; McDonald, S.A. Neurodevelopmental outcomes of extremely low birth weight infants <32 weeks' gestation between 1993 and 1998. *Pediatrics.* **2005**, *116*, 635-643.
13. Weintraub, Z.; Carmi, N.; Elouti, H.; Rumelt, S. The association between stage 3 or higher retinopathy of prematurity and other disorders of prematurity. *Can. J. Ophthalmol.* **2011**, *46*, 419-424.
14. Arnold, L.D. The cost-effectiveness of using banked donor milk in the neonatal intensive care unit: Prevention of necrotizing enterocolitis. *J Hum Lact.* **2002**, *18*, 172-177.
15. Braga, T.D.; da Silva, G.A.P.; Lira, P.I.C.; Lima, M.C. Necrotizing enterocolitis in very low weight newborns at a high risk neonatal unit in the northeast region of brazil (2003-2007). *Rev. Bras. Saude Matern. Infant.* **2012**, *12*, 127-133.
16. da Matta Aprile, M.; Feferbaum, R.; Andreassa, N.; Leone, C. Growth of very low birth weight infants fed with milk from a human milk bank selected according to the caloric and protein value. *Clinics.* **2010**, *65*, 751-756.
17. Hallstrom, M.; Koivisto, A.M.; Janas, M.; Tammela, O. Frequency of and risk factors for necrotizing enterocolitis in infants born before 33 weeks of gestation. *Acta Paediatr.* **2003**, *92*, 111-113.

18. Abily-Donval, L.; Pinto-Cardoso, G.; Chadie, A.; Guerrot, A.M.; Torre, S.; Rondeau, S.; Marret, S.; Perinatal Network of, H.-N. Comparison in outcomes at two-years of age of very preterm infants born in 2000, 2005 and 2010. *PLoS One.* **2015**, *10*, e0114567.
19. Beaino, G.; Khoshnood, B.; Kaminski, M.; Marret, S.; Pierrat, V.; Vieux, R.; Thiriez, G.; Matis, J.; Picaud, J.C.; Rozé, J.C., *et al.* Predictors of the risk of cognitive deficiency in very preterm infants: The epipage prospective cohort. *Acta Paediatr.* **2011**, *100*, 370-378.
20. Bigger, H.R.; Fogg, L.J.; Patel, A.; Johnson, T.; Engstrom, J.L.; Meier, P.P. Quality indicators for human milk use in very low-birthweight infants: Are we measuring what we should be measuring? *J. Perinatol.* **2014**, *34*, 287-291.
21. Corpeleijn, W.E.; Kouwenhoven, S.M.P.; Paap, M.C.; Van Vliet, I.; Scheerder, I.; Muizer, Y.; Helder, O.K.; Van Goudoever, J.B.; Vermeulen, M.J. Intake of own mother's milk during the first days of life is associated with decreased morbidity and mortality in very low birth weight infants during the first 60 days of life. *Neonatology.* **2012**, *102*, 276-281.
22. Marinelli, K.A.; Lussier, M.M.; Brownell, E.; Herson, V.C.; Hagadorn, J.I. The effect of a donor milk policy on the diet of very low birth weight infants. *J Hum Lact.* **2014**, *30*, 310-316.
23. Meinzen-Derr, J.; Poindexter, B.; Wrage, L.; Morrow, A.L.; Stoll, B.; Donovan, E.F. Role of human milk in extremely low birth weight infants' risk of necrotizing enterocolitis or death. *J. Perinatol.* **2009**, *29*, 57-62.
24. Nicholl, R.M.; Gamsu, H.R. Changes in growth and metabolism in very low birthweight infants fed with fortified breast milk. *Acta Paediatr.* **1999**, *88*, 1056-1061.
25. Amin, S.B.; Merle, K.S.; Orlando, M.S.; Dalzell, L.E.; Guillet, R. Brainstem maturation in premature infants as a function of enteral feeding type. *Pediatrics.* **2000**, *106*, 318-322.
26. Chan, G.M.; Borschel, M.W.; Jacobs, J.R. Effects of human milk or formula feeding on the growth, behavior, and protein status of preterm infants discharged from the newborn intensive care unit. *Am J Clin Nutr.* **1994**, *60*, 710-716.
27. Ganapathy, V.; Hay, J.W.; Kim, J. Analysis of necrotizing enterocolitis costs among extremely preterm infants fed exclusively human-milk based diet vs. Human-milk fortified with bovine-milk based supplements. *Value Health.* **2010**, *13*, A183-A184.
28. Ganapathy, V.; Hay, J.W.; Kim, J.H. Costs of necrotizing enterocolitis and cost-effectiveness of exclusively human milk-based products in feeding extremely premature infants. *Breastfeeding Med.* **2012**, *7*, 29-37.
29. Manzoni, P.; Lista, G.; Messner, H.; Cattani, S.; Rinaldi, M.; Pugni, L.; Decembrino, L.; Stolfi, I.; Gallo, E.; Stronati, M., *et al.* Fresh maternal milk feeding prevents retinopathy of prematurity: Data from two multicenter, randomized, placebo-controlled trials in preterm VLBW neonates in NICU. **2009,** Pediatric Academic Societies annual congress *abstract*.
30. Paolo, M. Fresh maternal milk feeding prevents retinopathy of prematurity: Data from two multicenter, randomized, placebo-controlled trials in preterm VLBW neonates in NICU. **2008,** Pediatric Academic Societies annual congress *abstract.*
31. Cocskun, Y.; Dalkan, C.; Yabas, O.; Demirel, O.; Bayar, E.; Sakarya, S.; Muftuotlu, T.; Ersanli, D.; Akman, I. The risk of retinopathy of prematurity (ROP) and comparing series IGF1 levels and clinical score system. *J. Matern. Fetal. Neonatal. Med.* **2016,** 29, S1,doi:10.1080/14767058.2016.1191212
32. Drane, D.L.; Logemann, J.A. A critical evaluation of the evidence on the association between type of infant feeding and cognitive development. *Paediatr. Perinat. Epidemiol.* **2000**, *14*, 349-356.
33. Dritsakou, K.; Liosis, G.; Valsami, G.; Polychronopoulos, E.; Skouroliakou, M. Improved outcomes of feeding low birth weight infants with predominantly raw human milk versus donor banked milk and formula. *J. Matern.-Fetal Neonatal Med.* **2016**, *29*, 1131-1138.
34. Dritsakou, K.; Liosis, G.; Valsami, G.; Polychronopoulos, E.; Souliotis, K.; Skouroliakou, M. Mother's breast milk supplemented with donor milk reduces hospital and health service usage costs in low-birthweight infants. *Midwifery.* **2016**, *40*, 109-113.

35. el-Mohandes, A.E.; Picard, M.B.; Simmens, S.J.; Keiser, J.F. Use of human milk in the intensive care nursery decreases the incidence of nosocomial sepsis. *J Perinatol.* **1997**, *17*, 130-134.
36. Morley, R.; Fewtrell, M.S.; Abbott, R.A.; Stephenson, T.; MacFadyen, U.; Lucas, A. Neurodevelopment in children born small for gestational age: A randomized trial of nutrient-enriched versus standard formula and comparison with a reference breastfed group. *Pediatrics.* **2004**, *113*, 515-521.
37. Hoque, M.M.; Ahmed, N.U.; Khan, F.H.; Jahan, R.; Yasmeen, H.N.; Chowdhury, M.A. Breastfeeding and cognitive development of children: Assessment at one year of age. *Mymensingh. Med. J.* **2012**, *21*, 316-321.
38. Morley, R.; Lucas, A. Randomized diet in the neonatal period and growth performance until 7.5- 8 y of age in preterm children. *Am. J. Clin. Nutr.* **2000**, *71*, 822-828.
39. Morley, R. Breast feeding and cognitive outcome in children born prematurely. *Adv. Exp. Med. Biol.* **2002**, *503*, 77-82.
40. Boyd, C.A.; Quigley, M.A.; Brocklehurst, P. Donor breast milk versus infant formula for preterm infants: Systematic review and meta-analysis. *Arch. Dis. Child. Fetal Neonatal Ed.* **2007**, *92*, F169-F175.
41. Elgen, I.; Sommerfelt, K.; Ellertsen, B. Cognitive performance in a low birth weight cohort at 5 and 11 years of age. *Pediatr. Neurol.* **2003**, *29*, 111-116.
42. Anderson, J.W.; Johnstone, B.M.; Remley, D.T. Breast-feeding and cognitive development: A meta-analysis. *Am. J. Clin. Nutr.* **1999**, *70*, 525-535.
43. Lucas, A.; Morley, R.; Cole, T.J.; Gore, S.M. A randomised multicentre study of human milk versus formula and later development in preterm infants. *Arch. Dis. Child. Fetal Neonatal Ed.* **1994**, 70, F141-146.
44. Colaizy, T.T.; Bartick, M.C.; Jegier, B.J.; Green, B.D.; Reinhold, A.G.; Schaefer, A.J.; Bogen, D.L.; Schwarz, E.B.; Stuebe, A.M.; Eunice Kennedy Shriver National Institute of Child, H., *et al.* Impact of optimized breastfeeding on the costs of necrotizing enterocolitis in extremely low birthweight infants. *J. Pediatr.* **2016**, *175*, 100-105.e2.
45. Mahon, J.; Claxton, L.; Wood, H. Modelling the cost-effectiveness of human milk and breastfeeding in preterm infants in the united kingdom. *Health Econ. Rev.* **2016**, *6*., 54
46. Abrams, S.A.; Schanler, R.J.; Lee, M.L.; Rechtman, D.J. Greater mortality and morbidity in extremely preterm infants fed a diet containing cow milk protein products. *Breastfeed. Med.* **2014**, *9*, 281-285.
47. Fang, J.L.; Sorita, A.; Carey, W.A.; Colby, C.E.; Hassan Murad, M.; Alahdab, F. Interventions to prevent retinopathy of prematurity: A meta-analysis. *Pediatrics.* **2016**, *137*.
48. McGuire, W.; Anthony, M.Y. Donor human milk versus formula for preventing necrotising enterocolitis in preterm infants: Systematic review. *Arch. Dis. Child. Fetal Neonatal Ed.* **2003**, *88*, F11-F14.
49. Sisk, P.M.; Lovelady, C.A.; Gruber, K.J.; Dillard, R.G.; O'Shea, T.M. Human milk consumption and full enteral feeding among infants who weigh </= 1250 grams. *Pediatrics.* **2008**, *121*, e1528-1533.
50. Smith, M.M.; Durkin, M.; Hinton, V.J.; Bellinger, D.; Kuhn, L. Influence of breastfeeding on cognitive outcomes at age 6-8 years: Follow-up of very low birth weight infants. *Am. J. Epidemiol.* **2003**, *158*, 1075-1082.
51. Gibertoni, D.; Corvaglia, L.; Vandini, S.; Rucci, P.; Savini, S.; Alessandroni, R.; Sansavini, A.; Fantini, M.P.; Faldella, G. Positive effect of human milk feeding during NICU hospitalization on 24 month neurodevelopment of very low birth weight infants: An italian cohort study. *PLoS ONE* **2015**, *10*, e0116552.
52. Roze, J.C.; Darmaun, D.; Boquien, C.Y.; Flamant, C.; Picaud, J.C.; Savagner, C.; Claris, O.; Lapillonne, A.; Mitanchez, D.; Branger, B., *et al.* The apparent breastfeeding paradox in very preterm infants: Relationship between breast feeding, early weight gain and

neurodevelopment based on results from two cohorts, epipage and lift. *BMJ Open* **2012**, *2*, e000834.

53. Adamkin, D.H. Mother's milk, feeding strategies, and lactoferrin to prevent necrotizing enterocolitis. *JPEN J. Parenter. Enteral Nutr.* **2012**, *36*, 25S-29S.
54. Behrman, D.; Broadfoot, M.; Buchanan, P.; Lamont, C.; Sachs, M. Early diet in preterm babies and later intelligence quotient. Surely study showed that breast milk is feed of choice for premature babies. *BMJ.* **1999**, *318*, 1625.
55. Chapman, D.J. Human milk dose in the first month is inversely associated with sepsis and nicu costs. *J. Hum. Lact.* **2013**, *29*, 339-340.
56. Rey, J. Breastfeeding and cognitive development. *Acta Paediatr.* **2003**, *92*, S11-S18.
57. Furman, L. Yes, human milk does reduce infection rates in very low birthweight infants. *Arch Dis Child Fetal Neonatal Ed.* **2006**, *91*, F78.
58. Raghuveer, T.S.; Belmont, J.M. Human milk intake and retinopathy of prematurity in extremely low birth weight infants. *Pediatrics.* **2008**, *122*, 686-687.
59. Verd, S.; Ginovart, G. Weight gain and bronchopulmonary dysplasia in human milk fed preterm infants. *J. Pediatr.* **2016**, *173*, 267.
60. Wight, N.E. Donor human milk versus formula for preventing necrotising enterocolitis in preterm infants: Systematic review. *J. Pediatr.* **2003**, *143*, 137-138.
61. Henderson, G.; Anthony, M.Y.; McGuire, W. Formula milk versus maternal breast milk for feeding preterm or low birth weight infants. *Cochrane Database Syst. Rev.* **2007**. 10.1002/14651858.CD002972.pub2
62. Bharwani, S.K.; Green, B.F.; Pezzullo, J.C.; Bharwani, S.S.; Bharwani, S.S.; Dhanireddy, R. Systematic review and meta-analysis of human milk intake and retinopathy of prematurity: A significant update. *J. Perinatol.* **2016**, *36*, 913-920.
63. Cacho, N.T.; Parker, L.A.; Neu, J. Necrotizing enterocolitis and human milk feeding: A systematic review. *Clin. Perinatol.* **2017**, *44*, 49-67.
64. De Silva, A.; Jones, P.W.; Spencer, S.A. Does human milk reduce infection rates in preterm infants? A systematic review. *Arch. Dis. Child. Fetal Neonatal Ed.* **2004**, *89*, F509-F513.
65. Koo, W.; Tank, S.; Martin, S.; Shi, R. Human milk and neurodevelopment in children with very low birth weight: A systematic review. *Nutr. J.* **2014**, *13*.
66. Lechner, B.E.; Vohr, B.R. Neurodevelopmental outcomes of preterm infants fed human milk: A systematic review. *Clin. Perinatol.* **2017**, *44*, 69-83.
67. Zhou, J.; Shukla, V.V.; John, D.; Chen, C. Human milk feeding as a protective factor for retinopathy of prematurity: A meta-analysis. *Pediatrics* **2015**, *136*, e1576-e1586.
68. Quigley, M.; McGuire, W. Formula versus donor breast milk for feeding preterm or low birth weight infants. *Cochrane Database Syst. Rev.* **2014**, Issue 4. Art. No.: CD002971. DOI: 10.1002/14651858.CD002971.pub3.
69. Johnson, T.J.; Patel, A.L.; Bigger, H.R.; Engstrom, J.L.; Meier, P.P. Economic benefits and costs of human milk feedings: A strategy to reduce the risk of prematurity-related morbidities in very-low-birth-weight infants. *Adv Nutr Res* **2014**, *5*, 207-212.
70. DiBiasie, A. Evidence-based review of retinopathy of prematurity prevention in vlbw and elbw infants. *Neonatal Netw.* **2006**, *25*, 393-403.
71. Kosloske, A.M. Breast milk decreases the risk of neonatal necrotizing enterocolitis. *Adv Nutr Res* **2001**, *10*, 123-137.
72. Collins, C.T.; Gibson, R.A.; Makrides, M.; McPhee, A.J.; Sullivan, T.R.; Davis, P.G.; Thio, M.; Simmer, K.; Rajadurai, V.S. The N3RO trial: A randomised controlled trial of docosahexaenoic acid to reduce bronchopulmonary dysplasia in preterm infants < 29 weeks' gestation. *BMC Pediatr.* **2016**, *16*, 72.
73. Unger, S.; Gibbins, S.; Zupancic, J.; O'Connor, D.L. Domino: Donor milk for improved neurodevelopmental outcomes. *BMC Pediatr.* **2014**, *14*.

74. Quigley, M.A.; Henderson, G.; Anthony, M.Y.; McGuire, W. Formula milk versus donor breast milk for feeding preterm or low birth weight infants. *Cochrane Database Syst Rev* **2007**.
75. Sánchez-Tamayo, T.; Espinosa Fernández, M.G.; Affumicato, L.; González López, M.; Fernández Romero, V.; Moreno Algarra, M.C.; Salguero García, E. Reduction in necrotising enterocolitis after implementing an evidence-based enteral nutrition protocol in very low birth weight newborns. *An. Pediatr.* **2016**, *85*, 291-299.

Study or Subgroup	Human milk/unpasteurised Events	Human milk/unpasteurised Total	PTF/pasteurised HM Events	PTF/pasteurised HM Total	Weight	Risk Ratio M-H, Random, 95% CI
**2.1.1 EHM vs EPTF; RTs**						
Cristofalo et al. 2013	0	29	4	24	100.0%	0.09 [0.01, 1.64]
**Subtotal (95% CI)**		**29**		**24**	**100.0%**	**0.09 [0.01, 1.64]**
Total events	0		4			
Heterogeneity: Not applicable						
Test for overall effect: Z = 1.62 (P = 0.10)						
**2.1.2 EHM vs EPTF; Observational**						
Spiegler et al. 2016	1	212	5	232	100.0%	0.22 [0.03, 1.86]
**Subtotal (95% CI)**		**212**		**232**	**100.0%**	**0.22 [0.03, 1.86]**
Total events	1		5			
Heterogeneity: Not applicable						
Test for overall effect: Z = 1.39 (P = 0.16)						
**2.1.3 Any HM vs EPTF; Observational**						
Chowning et al 2016	0	71	6	76	23.9%	0.08 [0.00, 1.43]
Schanler et al. 1999	0	62	3	46	23.0%	0.11 [0.01, 2.01]
Spiegler et al. 2016	17	933	5	232	53.1%	0.85 [0.32, 2.27]
**Subtotal (95% CI)**		**1066**		**354**	**100.0%**	**0.30 [0.05, 1.76]**
Total events	17		14			
Heterogeneity: Tau² = 1.28; Chi² = 4.01, df = 2 (P = 0.13); I² = 50%						
Test for overall effect: Z = 1.33 (P = 0.18)						
**2.1.4 Higher vs lower dose HM; RTs**						
Corpeleijn et al. 2016	4	183	5	190	52.9%	0.83 [0.23, 3.04]
Sullivan et al. 2010	2	138	7	69	47.1%	0.14 [0.03, 0.67]
**Subtotal (95% CI)**		**321**		**259**	**100.0%**	**0.36 [0.06, 2.04]**
Total events	6		12			
Heterogeneity: Tau² = 1.02; Chi² = 2.93, df = 1 (P = 0.09); I² = 66%						
Test for overall effect: Z = 1.15 (P = 0.25)						
**2.1.5 Higher vs lower dose HM; Observational**						
Bishop et al. 2010	5	152	13	179	19.1%	0.45 [0.17, 1.24]
Chowning et al 2016	6	290	11	260	20.2%	0.49 [0.18, 1.30]
Ginovart et al 2016	7	114	4	72	13.7%	1.11 [0.34, 3.64]
Sisk et al 2017	10	438	8	113	23.7%	0.32 [0.13, 0.80]
Spiegler et al. 2016	1	212	17	933	4.8%	0.26 [0.03, 1.93]
Verd et al. 2015	10	148	5	53	18.5%	0.72 [0.26, 2.00]
**Subtotal (95% CI)**		**1354**		**1610**	**100.0%**	**0.51 [0.33, 0.79]**
Total events	39		58			
Heterogeneity: Tau² = 0.00; Chi² = 3.52, df = 5 (P = 0.62); I² = 0%						
Test for overall effect: Z = 3.01 (P = 0.003)						
**2.1.6 Unpasteurised vs pasteurised HM; RTs**						
Cossey et al. 2013	0	151	4	152	100.0%	0.11 [0.01, 2.06]
**Subtotal (95% CI)**		**151**		**152**	**100.0%**	**0.11 [0.01, 2.06]**
Total events	0		4			
Heterogeneity: Not applicable						
Test for overall effect: Z = 1.47 (P = 0.14)						
**2.1.7 Unpasteurised vs pasteurised HM; Observational**						
Giuliani et al. 2012	0	46	1	46	38.2%	0.33 [0.01, 7.98]
Sisk et al 2017	9	299	1	139	61.8%	4.18 [0.54, 32.70]
**Subtotal (95% CI)**		**345**		**185**	**100.0%**	**1.59 [0.14, 17.85]**
Total events	9		2			
Heterogeneity: Tau² = 1.36; Chi² = 1.73, df = 1 (P = 0.19); I² = 42%						
Test for overall effect: Z = 0.38 (P = 0.71)						

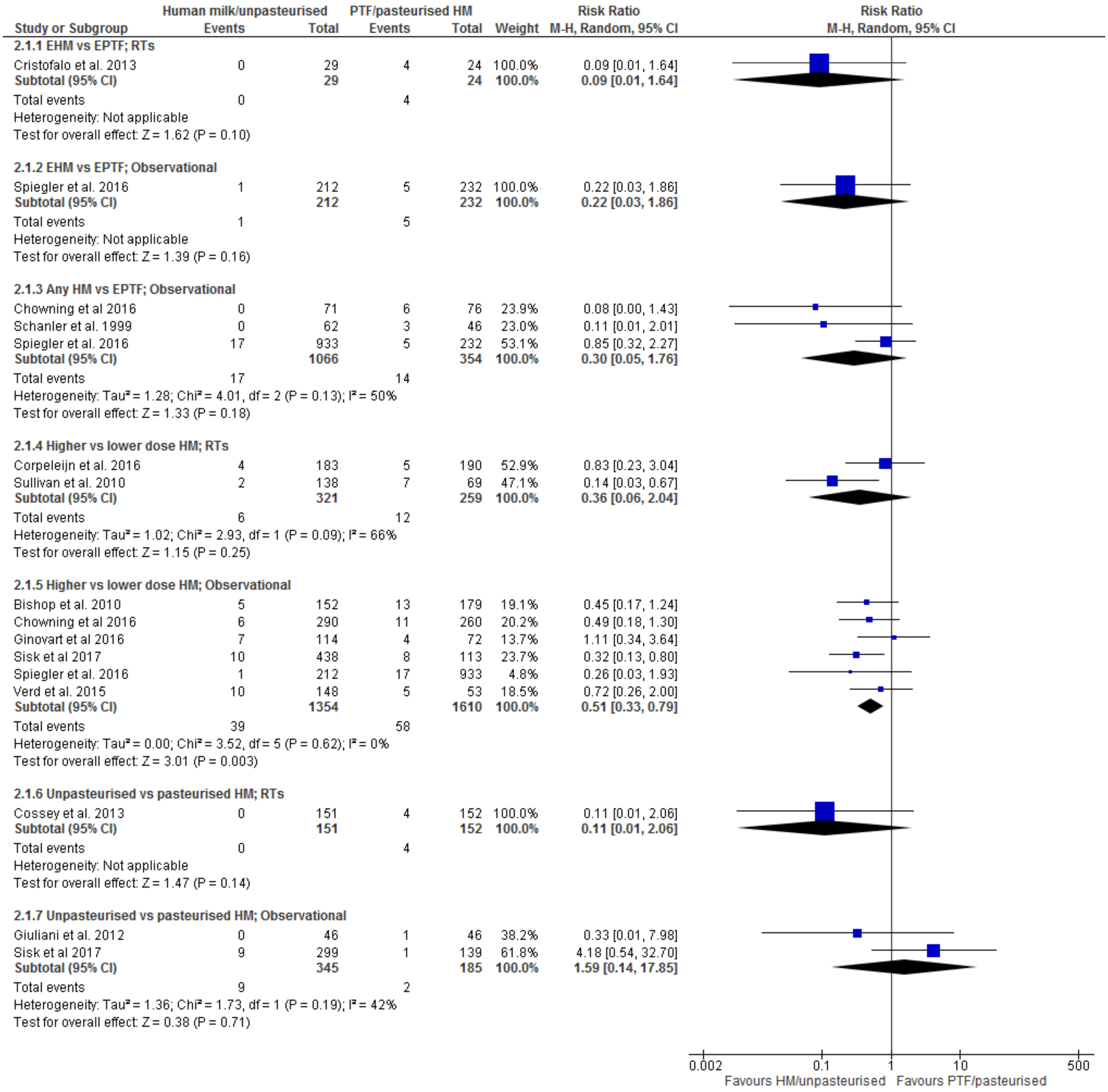

Figure S2. Forest plot of relative risk for the association of human milk and severe necrotising enterocolitis

Table S3. Any necrotising enterocolitis: summary of findings

Comparisons	Anticipated absolute effects* (95% CI)		Relative effect (95% CI)	№ of participants (studies)	Certainty of the evidence (GRADE)
	Risk with preterm formula/ pasteurised human milk	Risk with human milk/ unpasteurised human milk			
**Necrotising enterocolitis - Exclusive human milk vs exclusive preterm formula; RTs**	Study population		RR 0.17 (0.02 to 1.32)	53 (1 RCT)	⊕⊕⊖⊖ LOW [1]
	208 per 1,000	35 per 1,000 (4 to 275)			
**Necrotising enterocolitis - Exclusive human milk vs exclusive preterm formula; Non-RT & Observational**	Study population		RR 0.22 (0.09 to 0.54)	993 (3 observational studies)	⊕⊕⊕⊖ MODERATE
	55 per 1,000	12 per 1,000 (5 to 30)			
**Necrotising enterocolitis - Any human milk vs exclusive preterm formula; Observational**	Study population		RR 0.51 (0.35 to 0.76)	3783 (9 observational studies)	⊕⊕⊕⊖ MODERATE
	73 per 1,000	37 per 1,000 (26 to 56)			
**Necrotising enterocolitis - Higher vs lower dose human milk intake; RTs**	Study population		RR 0.54 (0.28 to 1.02)	1116 (4 RCTs)	⊕⊕⊕⊖ MODERATE [2 3]
	94 per 1,000	51 per 1,000 (26 to 96)			
**Necrotising enterocolitis - Higher vs lower dose human milk intake; Observational**	Study population		RR 0.53 (0.42 to 0.67)	8778 (22 observational studies)	⊕⊕⊕⊖ MODERATE
	80 per 1,000	42 per 1,000 (34 to 54)			
**Necrotising enterocolitis - Unpasteurised vs pasteurised human milk (MOM or donor); RTs**	Study population		RR 1.45 (0.64 to 3.30)	303 (1 RCT)	⊕⊕⊖⊖ LOW [4]
	59 per 1,000	86 per 1,000 (38 to 195)			
**Necrotising enterocolitis - Unpasteurised vs pasteurised human milk (MOM or donor); Observational**	Study population		RR 1.28 (0.68 to 2.43)	1894 (6 observational studies)	⊕⊖⊖⊖ VERY LOW [5]
	37 per 1,000	47 per 1,000 (25 to 90)			
*The risk in the intervention group (and its 95% confidence interval) is based on the assumed risk in the comparison group and the relative effect of the intervention (and its 95% CI). CI: Confidence interval; RR: Risk ratio; OR: Odds ratio; RT: Randomised Trial					
GRADE Working Group grades of evidence<br>High certainty: We are very confident that the true effect lies close to that of the estimate of the effect<br>Moderate certainty: We are moderately confident in the effect estimate: The true effect is likely to be close to the estimate of the effect, but there is a possibility that it is substantially different<br>Low certainty: Our confidence in the effect estimate is limited: The true effect may be substantially different from the estimate of the effect<br>Very low certainty: We have very little confidence in the effect estimate: The true effect is likely to be substantially different from the estimate of effect					

Footnotes: [1]1 study, wide CIs, small sample size; [2]Sullivan was not blinded, however, the outcome is objective and unlikely to be biased by this, hence not downgraded; [3]Moderate heterogeneity; [4]1 study, wide Cis; [5]Wide CIs

Table S4. Severe necrotising enterocolitis: summary of findings

Comparisons	Anticipated absolute effects* (95% CI)		Relative effect (95% CI)	№ of participants (studies)	Certainty of the evidence (GRADE)
	**Risk with preterm formula/ pasteurised human milk**	**Risk with human milk/ unpasteurised human milk**			
**NEC surgery - EHM vs EPTF; RTs**	Study population		RR 0.09 (0.01 to 1.64)	53 (1 RT)	⊕⊕⊖⊖ LOW [1]
	167 per 1,000	15 per 1,000 (2 to 273)			
**NEC surgery - EHM vs EPTF; Observational**	Study population		RR 0.22 (0.03 to 1.86)	444 (1 observational study)	⊕⊖⊖⊖ VERY LOW [1]
	22 per 1,000	5 per 1,000 (1 to 40)			
**NEC surgery - Any HM vs EPTF; Observational**	Study population		RR 0.30 (0.05 to 1.76)	1420 (3 observational studies)	⊕⊖⊖⊖ VERY LOW [2]
	40 per 1,000	12 per 1,000 (2 to 70)			
**NEC surgery - Higher vs lower dose HM; RTs**	Study population		RR 0.36 (0.06 to 2.04)	580 (2 RCTs)	⊕⊕⊖⊖ LOW [2 3]
	46 per 1,000	17 per 1,000 (3 to 95)			
**NEC surgery - Higher vs lower dose HM; Observational**	Study population		RR 0.51 (0.33 to 0.79)	2964 (6 observational studies)	⊕⊕⊕⊖ MODERATE
	36 per 1,000	18 per 1,000 (12 to 28)			
**NEC surgery - Unpasteurised vs pasteurised HM; RTs**	Study population		RR 0.11 (0.01 to 2.06)	303 (1 RCT)	⊕⊕⊖⊖ LOW [1]
	26 per 1,000	3 per 1,000 (0 to 54)			
**NEC surgery - Unpasteurised vs pasteurised HM; Observational**	Study population		RR 1.59 (0.14 to 17.85)	530 (2 observational studies)	⊕⊖⊖⊖ VERY LOW[3]
	11 per 1,000	17 per 1,000 (2 to 193)			

*The risk in the intervention group (and its 95% confidence interval) is based on the assumed risk in the comparison group and the relative effect of the intervention (and its 95% CI).
CI: Confidence interval; RR: Risk ratio; OR: Odds ratio;

GRADE Working Group grades of evidence
High certainty: We are very confident that the true effect lies close to that of the estimate of the effect
Moderate certainty: We are moderately confident in the effect estimate: The true effect is likely to be close to the estimate of the effect, but there is a possibility that it is substantially different
Low certainty: Our confidence in the effect estimate is limited: The true effect may be substantially different from the estimate of the effect
Very low certainty: We have very little confidence in the effect estimate: The true effect is likely to be substantially different from the estimate of effect

Footnotes: [1]Single study, wide CIs; [2]Moderate heterogeneity; [3]Wide CI

Table S5. Late onset sepsis: summary of findings

Comparisons	Anticipated absolute effects* (95% CI)		Relative effect (95% CI)	№ of participants (studies)	Certainty of the evidence (GRADE)
	Risk with preterm formula/ pasteurised human milk	Risk with human milk/ unpasteurised human milk			
**Late onset sepsis - Exclusive human milk vs exclusive preterm formula: RT**	Study population		RR 0.70 (0.47 to 1.03)	53 (1 RCT)	⊕⊕⊖⊖ LOW [1]
	792 per 1,000	554 per 1,000 (372 to 815)			
**Late onset sepsis - Exclusive human milk vs exclusive preterm formula; Non-RT and Observational**	Study population		RR 0.71 (0.49 to 1.05)	776 (3 observational studies)	⊕⊕⊖⊖ LOW
	174 per 1,000	123 per 1,000 (85 to 183)			
**Late onset sepsis - Any human milk vs exclusive preterm formula; Observational**	Study population		RR 0.95 (0.67 to 1.34)	2497 (8 observational studies)	⊕⊖⊖⊖ VERY LOW [2 3]
	301 per 1,000	286 per 1,000 (202 to 404)			
**Late onset sepsis - Higher vs lower dose human milk intake; RTs**	Study population		RR 1.07 (0.89 to 1.28)	1186 (4 RCTs)	⊕⊕⊕⊖ MODERATE [3]
	276 per 1,000	295 per 1,000 (245 to 353)			
**Late onset sepsis - Higher vs lower dose human milk intake; Observational**	Study population		RR 0.71 (0.56 to 0.90)	6521 (18 observational studies)	⊕⊖⊖⊖ VERY LOW [2]
	230 per 1,000	163 per 1,000 (129 to 207)			
**Late onset sepsis - Unpasteurised vs pasteurised human milk (MOM or donor); RT**	Study population		RR 0.71 (0.43 to 1.18)	303 (1 RCT)	⊕⊕⊕⊖ MODERATE [4]
	204 per 1,000	145 per 1,000 (88 to 241)			
**Late onset sepsis - Unpasteurised vs pasteurised human milk (MOM or donor); Observational**	Study population		RR 1.05 (0.86 to 1.27)	1875 (5 observational studies)	⊕⊖⊖⊖ VERY LOW [5]
	258 per 1,000	271 per 1,000 (222 to 328)			

*The risk in the intervention group (and its 95% confidence interval) is based on the assumed risk in the comparison group and the relative effect of the intervention (and its 95% CI).
CI: Confidence interval; RR: Risk ratio; OR: Odds ratio;

GRADE Working Group grades of evidence
High certainty: We are very confident that the true effect lies close to that of the estimate of the effect
Moderate certainty: We are moderately confident in the effect estimate: The true effect is likely to be close to the estimate of the effect, but there is a possibility that it is substantially different
Low certainty: Our confidence in the effect estimate is limited: The true effect may be substantially different from the estimate of the effect
Very low certainty: We have very little confidence in the effect estimate: The true effect is likely to be substantially different from the estimate of effect

Footnotes: [1]Only 1 study, wide CIs; [2]Substantial heterogeneity; [3.]Wide CI; [4.]1 study only; [5]Moderate heterogeneity

Table S6. Bronchopulmonary dysplasia: summary of findings

Comparisons	Anticipated absolute effects* (95% CI)		Relative effect (95% CI)	№ of participants (studies)	Certainty of the evidence (GRADE)
	Risk with preterm formula/ pasteurised human milk	Risk with human milk/ unpasteurised human milk			
**Bronchopulmonary dysplasia - Exclusive human milk vs exclusive preterm formula: Observational**	Study population		RR 0.94 (0.26 to 3.41)	706 (2 observational studies)	⊕⊖⊖⊖ VERY LOW [1 2]
	197 per 1,000	185 per 1,000 (51 to 672)			
**Bronchopulmonary dysplasia - Any human milk vs exclusive preterm formula; Observational**	Study population		RR 1.02 (0.83 to 1.27)	3703 (6 observational studies)	⊕⊖⊖⊖ VERY LOW [2 3]
	345 per 1,000	352 per 1,000 (286 to 438)			
**Bronchopulmonary dysplasia - Higher vs lower dose human milk intake: RTs**	Study population		RR 0.95 (0.73 to 1.25)	1075 (4 RCTs)	⊕⊕⊖⊖ LOW [2 3]
	263 per 1,000	250 per 1,000 (192 to 328)			
**Bronchopulmonary dysplasia - Higher vs lower dose human milk intake; Observational**	Study population		RR 0.84 (0.73 to 0.96)	7023 (18 observational studies)	⊕⊖⊖⊖ VERY LOW [3]
	305 per 1,000	256 per 1,000 (223 to 293)			
**Bronchopulmonary dysplasia - Unpasteurised vs pasteurised human milk (MOM or donor); RTs**	Study population		RR 0.69 (0.43 to 1.10)	303 (1 RCT)	⊕⊕⊖⊖ LOW [4]
	230 per 1,000	159 per 1,000 (99 to 253)			
**Bronchopulmonary dysplasia - Unpasteurised vs pasteurised human milk (MOM or donor); Observational**	Study population		RR 1.01 (0.72 to 1.43)	1644 (5 observational studies)	⊕⊖⊖⊖ VERY LOW [2]
	203 per 1,000	205 per 1,000 (146 to 290)			
*The risk in the intervention group (and its 95% confidence interval) is based on the assumed risk in the comparison group and the relative effect of the intervention (and its 95% CI). CI: Confidence interval; RR: Risk ratio; OR: Odds ratio;					
GRADE Working Group grades of evidence<br>High certainty: We are very confident that the true effect lies close to that of the estimate of the effect<br>Moderate certainty: We are moderately confident in the effect estimate: The true effect is likely to be close to the estimate of the effect, but there is a possibility that it is substantially different<br>Low certainty: Our confidence in the effect estimate is limited: The true effect may be substantially different from the estimate of the effect<br>Very low certainty: We have very little confidence in the effect estimate: The true effect is likely to be substantially different from the estimate of effect					

Footnotes: [1]Substantial heterogeneity; [2]Wide CI; [3]Moderate heterogeneity; [4]Only 1 study, wide CI

Table S7. Retinopathy of prematurity: summary of findings

Comparison	Anticipated absolute effects* (95% CI)		Relative effect (95% CI)	№ of participants (studies)	Certainty of the evidence (GRADE)
	Risk with preterm formula/ pasteurised human milk	Risk with human milk/ Unpasteurised human milk			
**Retinopathy of prematurity - Exclusive human milk vs exclusive preterm formula; RTs**	Study population		RR 1.32 (0.50 to 3.52)	53 (1 RCT)	⊕⊕⊖⊖ LOW [1]
	208 per 1,000	275 per 1,000 (104 to 733)			
**Retinopathy of prematurity - Exclusive human milk vs exclusive preterm formula; Observational**	Study population		RR 0.65 (0.31 to 1.34)	1256 (4 observational studies)	⊕⊖⊖⊖ VERY LOW [2 3]
	329 per 1,000	214 per 1,000 (102 to 441)			
**Retinopathy of prematurity - Any human milk vs exclusive preterm formula; Observational**	Study population		RR 1.08 (0.79 to 1.48)	3576 (6 observational studies)	⊕⊖⊖⊖ VERY LOW [2 3]
	286 per 1,000	309 per 1,000 (226 to 423)			
**Retinopathy of prematurity - Higher vs lower dose human milk intake; RTs**	Study population		RR 1.14 (0.86 to 1.50)	1071 (4 RCTs)	⊕⊕⊕⊖ MODERATE [3]
	119 per 1,000	136 per 1,000 (103 to 179)			
**Retinopathy of prematurity - Higher vs lower dose human milk intake; Observational**	Study population		RR 0.82 (0.70 to 0.96)	6302 (18 observational studies)	⊕⊖⊖⊖ VERY LOW [4]
	210 per 1,000	172 per 1,000 (147 to 202)			
**Retinopathy of prematurity - Unpasteurised vs pasteurised human milk (MOM or donor); RTs**	Study population		RR 0.89 (0.35 to 2.26)	303 (1 RCT)	⊕⊕⊖⊖ LOW [5]
	59 per 1,000	53 per 1,000 (21 to 134)			
**Retinopathy of prematurity - Unpasteurised vs pasteurised human milk (MOM or donor); Observational**	Study population		RR 0.89 (0.33 to 2.38)	681 (3 observational studies)	⊕⊖⊖⊖ VERY LOW [2 3]
	117 per 1,000	104 per 1,000 (38 to 277)			
*The risk in the intervention group (and its 95% confidence interval) is based on the assumed risk in the comparison group and the relative effect of the intervention (and its 95% CI). CI: Confidence interval; RR: Risk ratio; OR: Odds ratio;					
GRADE Working Group grades of evidence<br>High certainty: We are very confident that the true effect lies close to that of the estimate of the effect<br>Moderate certainty: We are moderately confident in the effect estimate: The true effect is likely to be close to the estimate of the effect, but there is a possibility that it is substantially different<br>Low certainty: Our confidence in the effect estimate is limited: The true effect may be substantially different from the estimate of the effect<br>Very low certainty: We have very little confidence in the effect estimate: The true effect is likely to be substantially different from the estimate of effect					

Footnotes: [1]Wide CIs; only 1 study; small sample; [2]Substantial heterogeneity; [3]Wide CI; [4]Moderate heterogeneity; [5]Only 1 study, wide CIs.

Study or Subgroup	Human milk/unpasteurised Events	Human milk/unpasteurised Total	PTF/pasteurised HM Events	PTF/pasteurised HM Total	Weight	Risk Ratio M-H, Random, 95% CI
**4.1.1 EHM vs EPTF; Observational**						
Hylander 2001 (1)	0	17	9	74	13.3%	0.22 [0.01, 3.60]
Manzoni 2013	4	314	22	184	40.9%	0.11 [0.04, 0.30]
Spiegler 2016	7	203	16	220	45.8%	0.47 [0.20, 1.13]
**Subtotal (95% CI)**		**534**		**478**	**100.0%**	**0.23 [0.07, 0.73]**
Total events	11		47			
Heterogeneity: Tau² = 0.56; Chi² = 4.69, df = 2 (P = 0.10); I² = 57%						
Test for overall effect: Z = 2.49 (P = 0.01)						
**4.1.2 Any HM vs EPTF; Observational**						
Heller 2007 (2)	130	788	33	269	41.4%	1.34 [0.94, 1.92]
Hylander 2001 (3)	6	100	9	74	22.9%	0.49 [0.18, 1.33]
Spiegler 2016	49	1102	16	220	35.6%	0.61 [0.35, 1.05]
**Subtotal (95% CI)**		**1990**		**563**	**100.0%**	**0.81 [0.42, 1.56]**
Total events	185		58			
Heterogeneity: Tau² = 0.24; Chi² = 7.76, df = 2 (P = 0.02); I² = 74%						
Test for overall effect: Z = 0.64 (P = 0.52)						
**4.1.3 Higher vs lower dose HM; RTs**						
O'Connor 2016	7	181	8	182	31.9%	0.88 [0.33, 2.38]
Schanler 2005 (4)	15	81	13	92	68.1%	1.31 [0.66, 2.59]
**Subtotal (95% CI)**		**262**		**274**	**100.0%**	**1.15 [0.66, 2.02]**
Total events	22		21			
Heterogeneity: Tau² = 0.00; Chi² = 0.42, df = 1 (P = 0.52); I² = 0%						
Test for overall effect: Z = 0.50 (P = 0.62)						
**4.1.4 Higher vs lower dose HM; Observational**						
Alshaikh 2015	14	254	6	189	8.9%	1.74 [0.68, 4.43]
Colacci 2017	2	39	4	46	3.4%	0.59 [0.11, 3.05]
Ginovart 2016	1	114	3	72	1.9%	0.21 [0.02, 1.99]
Hair 2016	43	819	69	768	25.5%	0.58 [0.40, 0.84]
Hylander 2001 (5)	0	45	2	16	1.1%	0.07 [0.00, 1.46]
Kreissl 2017	5	150	20	150	8.7%	0.25 [0.10, 0.65]
Maayan-Metzger 2012	3	188	7	172	5.0%	0.39 [0.10, 1.49]
Madore 2017	2	29	0	25	1.1%	4.33 [0.22, 86.22]
Schanler 2005 (6)	4	70	13	92	7.2%	0.40 [0.14, 1.19]
Sisk 2007	9	112	2	20	4.3%	0.80 [0.19, 3.45]
Sisk 2017 (7)	32	438	9	113	13.3%	0.92 [0.45, 1.87]
Spiegler 2016 (8)	7	203	42	899	11.6%	0.74 [0.34, 1.62]
Verd 2015 (9)	11	148	5	53	8.0%	0.79 [0.29, 2.16]
**Subtotal (95% CI)**		**2609**		**2615**	**100.0%**	**0.63 [0.46, 0.87]**
Total events	133		182			
Heterogeneity: Tau² = 0.07; Chi² = 15.44, df = 12 (P = 0.22); I² = 22%						
Test for overall effect: Z = 2.80 (P = 0.005)						
**4.1.5 Unpasteurised vs pasteurised HM; RTs**						
Cossey 2013	8	151	9	152	100.0%	0.89 [0.35, 2.26]
**Subtotal (95% CI)**		**151**		**152**	**100.0%**	**0.89 [0.35, 2.26]**
Total events	8		9			
Heterogeneity: Not applicable						
Test for overall effect: Z = 0.24 (P = 0.81)						
**4.1.6 Unpasteurised vs pasteurised: Observational**						
Schanler 2005 (10)	4	70	15	81	48.6%	0.31 [0.11, 0.89]
Sisk 2017 (11)	26	299	6	139	51.4%	2.01 [0.85, 4.78]
**Subtotal (95% CI)**		**369**		**220**	**100.0%**	**0.81 [0.13, 5.08]**
Total events	30		21			
Heterogeneity: Tau² = 1.52; Chi² = 7.26, df = 1 (P = 0.007); I² = 86%						
Test for overall effect: Z = 0.23 (P = 0.82)						

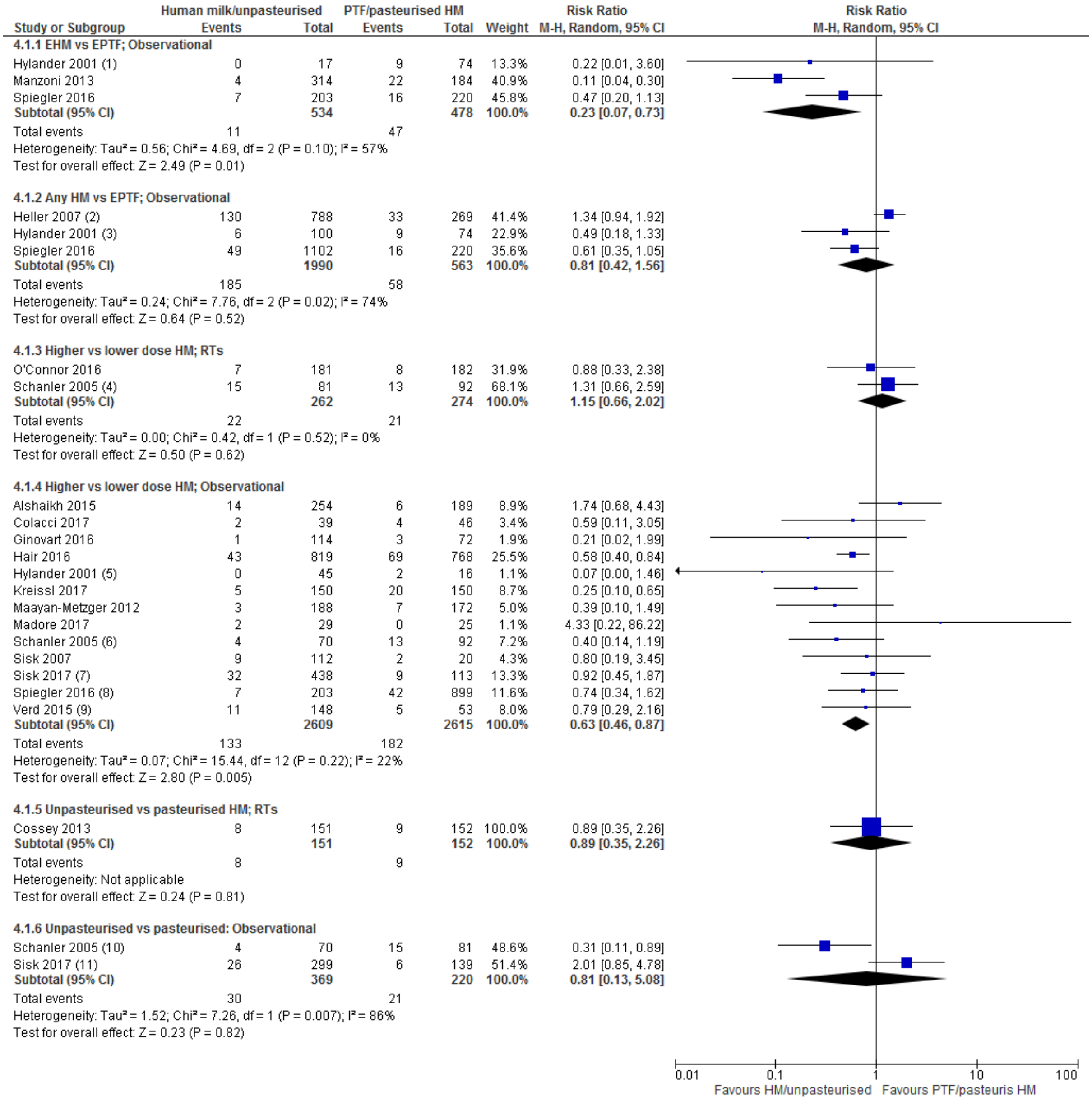

Footnotes
(1) Group '100% HM' compared with EPTF group
(2) Incidence calculated from odds ratio
(3) Groups 0-19%, 20-79%, 80-99% and 100% were collapsed for 'any human milk' and cf EPTF
(4) Groups 'PDM'(higher dose) cf group 'PTF as supplement to MOM" (lower dose)
(5) Group 0-19% (lower dose HM) compared with group 80 -99% and group 100% (higher dose HM)
(6) Group MOM (high dose) cf wtih group PTF (low dose). MOM group not randomised
(7) Groups '≥50% MOM' and '≥50% PDHM' combined for high dose and cf '≥50% EPTF'
(8) EHM = higher dose HM; mixed feeding = lower dose HM; grande 3/4
(9) Data derived from inverse of 'no ROP'; EHM = higher dose HM; mixed feeding = lower dose HM
(10) MOM non-randomised
(11) Group '≥50% MOM' cf '≥50% PDHM'

Figure S3. Forest plot of relative risk for the association between human milk and severe retinopathy of prematurity

Table S8. Severe retinopathy of prematurity: summary of findings

Comparisons	Anticipated absolute effects* (95% CI)		Relative effect (95% CI)	№ of participants (studies)	Certainty of the evidence (GRADE)
	Risk with preterm formula/ pasteurised human milk	Risk with human milk/ Unpasteurised human milk			
**Severe ROP - EHM vs EPTF; Observational**	Study population		RR 0.23 (0.07 to 0.73)	1012 (3 observational studies)	⊕⊕⊖⊖ LOW [1]
	98 per 1,000	23 per 1,000 (7 to 72)			
**Severe ROP - Any HM vs EPTF; Observational**	Study population		RR 0.81 (0.42 to 1.56)	2553 (3 observational studies)	⊕⊖⊖⊖ VERY LOW 1
	103 per 1,000	83 per 1,000 (43 to 161)			
**Severe ROP - Higher vs lower dose HM; RTs**	Study population		RR 1.15 (0.66 to 2.02)	536 (2 RCTs)	⊕⊕⊖⊖ LOW [2]
	77 per 1,000	88 per 1,000 (51 to 155)			
**Severe ROP - Higher vs lower dose HM; Observational**	Study population		RR 0.63 (0.46 to 0.87)	5224 (13 observational studies)	⊕⊕⊖⊖ LOW
	70 per 1,000	44 per 1,000 (32 to 61)			
**Severe ROP - Unpasteurised vs pasteurised HM; RTs**	Study population		RR 0.89 (0.35 to 2.26)	303 (1 RCT)	⊕⊕⊕⊕ HIGH
	59 per 1,000	53 per 1,000 (21 to 134)			
**Severe ROP - Unpasteurised vs pasteurised: Observational**	Study population		RR 0.81 (0.13 to 5.08)	589 (2 observational studies)	⊕⊖⊖⊖ VERY LOW [1]
	95 per 1,000	77 per 1,000 (12 to 485)			
***The risk in the intervention group (and its 95% confidence interval) is based on the assumed risk in the comparison group and the relative effect of the intervention (and its 95% CI).**<br><br>**CI: Confidence interval; RR: Risk ratio; OR: Odds ratio;**					
**GRADE Working Group grades of evidence**<br>**High certainty: We are very confident that the true effect lies close to that of the estimate of the effect**<br>**Moderate certainty: We are moderately confident in the effect estimate: The true effect is likely to be close to the estimate of the effect, but there is a possibility that it is substantially different**<br>**Low certainty: Our confidence in the effect estimate is limited: The true effect may be substantially different from the estimate of the effect**<br>**Very low certainty: We have very little confidence in the effect estimate: The true effect is likely to be substantially different from the estimate of effect**					

Footnotes: [1]Substantial heterogeneity; [2]Wide CIs, only 2 studies; [3]Cossey et al 2013 did not blind participants and personnel, however not downgraded for this as outcome is objective, outcome assessors were blinded, and bias is unlikely.

Table S9. Neurodevelopment: summary of findings

Outcomes	Anticipated absolute effects* (95% CI)		Relative effect (95% CI)	№ of participants (studies)	Certainty of the evidence (GRADE)
	Risk with preterm formula	Risk with human milk			
**Neurodevelopment – Cognition <18 months: Any human milk vs exclusive preterm formula; Observational**	The mean neurodevelopment – Cognition <18 months: Any human milk vs exclusive preterm formula; Observational was 0	MD 9 higher (1.42 higher to 16.58 higher)	-	39 (1 observational study)	⊕⊖⊖⊖ VERY LOW [1]
**Neurodevelopment – Cognition 18 to <36 months: Any human milk vs exclusive preterm formula; observational**	The mean neurodevelopment – Cognition 18 to <36 months: Any human milk vs exclusive preterm formula; observational was 0	MD 2.01 higher (1.35 lower to 5.36 higher)	-	1744 (3 observational studies)	⊕⊖⊖⊖ VERY LOW [2]
**Neurodevelopment – Cognition <18 months: Higher vs lower dose human milk; Observational**	The mean neurodevelopment – Cognition <18 months: Higher vs lower dose human milk; Observational was 0	MD 0.67 higher (2.68 lower to 4.03 higher)	-	684 (5 observational studies)	⊕⊖⊖⊖ VERY LOW [2]
**Neurodevelopment – Cognition 18 to <36 months: Higher vs lower dose human milk; RT**	The mean neurodevelopment – Cognition 18 to <36 months: Higher vs lower dose human milk; RT was 0	MD 1.6 lower (5.95 lower to 2.75 higher)	-	299 (1 RCT)	⊕⊕⊕⊖ MODERATE [3]
**Neurodevelopment – Cognition 18 to 36 months: Higher vs lower dose human milk; observational**	The mean neurodevelopment – Cognition 18 to 36 months: Higher vs lower dose human milk; observational was 0	MD 0.59 lower (3.41 lower to 2.24 higher)	-	722 (4 observational studies)	⊕⊖⊖⊖ VERY LOW [2]
**Neurodevelopment – Cognition >3 years; Higher vs lower dose human milk; Observational**	The mean neurodevelopment – Cognition >3 years; Higher vs lower dose human milk; Observational was 0	MD 6.4 higher (5.8 lower to 18.6 higher)	-	18 (1 observational study)	⊕⊖⊖⊖ VERY LOW [1]
**Neurodevelopment – Motor 18 to <36 months: Any human milk vs exclusive preterm formula; Observational**	The mean neurodevelopment – Motor 18 to <36 months: Any human milk vs exclusive preterm formula; Observational was 0	MD 0.8 lower (6.02 lower to 4.42 higher)	-	1744 (3 observational studies)	⊕⊖⊖⊖ VERY LOW [2,3]
**Neurodevelopment – Motor 18 to <36 months: Higher vs lower dose human milk; RT**	The mean neurodevelopment – Motor: Higher vs lower dose human milk; RT was 0	MD 2.2 lower (6.42 lower to 2.02 higher)	-	299 (1 RCT)	⊕⊕⊕⊖ MODERATE [1]
**Neurodevelopment – Motor<18 months: Higher vs lower dose human milk; Observational**	The mean neurodevelopment – Motor<18 months: Higher vs lower dose human milk; Observational was 0	MD 0.33 lower (4.8 lower to 4.14 higher)	-	684 (5 observational studies)	⊕⊖⊖⊖ VERY LOW [2,4]

Outcomes	Anticipated absolute effects* (95% CI)		Relative effect (95% CI)	№ of participants (studies)	Certainty of the evidence (GRADE)
	Risk with preterm formula	Risk with human milk			
**Neurodevelopment – Motor 18 to <36 months: Higher vs lower dose human milk; observational**	The mean neurodevelopment – Motor 18 to <36 months: Higher vs lower dose human milk; observational was 0	MD 1.94 lower (4.78 lower to 0.9 higher)	-	722 (4 observational studies)	⊕⊖⊖⊖ VERY LOW [2]
*The risk in the intervention group (and its 95% confidence interval) is based on the assumed risk in the comparison group and the relative effect of the intervention (and its 95% CI).<br><br>CI: Confidence interval; RR: Risk ratio; OR: Odds ratio;					
GRADE Working Group grades of evidence<br>High certainty: We are very confident that the true effect lies close to that of the estimate of the effect<br>Moderate certainty: We are moderately confident in the effect estimate: The true effect is likely to be close to the estimate of the effect, but there is a possibility that it is substantially different<br>Low certainty: Our confidence in the effect estimate is limited: The true effect may be substantially different from the estimate of the effect<br>Very low certainty: We have very little confidence in the effect estimate: The true effect is likely to be substantially different from the estimate of effect					

Footnotes: [1]Only 1 study; [2]Wide Cis; [3]Only 1 study, wide Cis; [4]Moderate heterogeneity
